# ZNT5-6 and ZNT7 play an integral role in protein *N*-glycosylation by supplying Zn^2+^ to Golgi α-mannosidase II

**DOI:** 10.1016/j.jbc.2024.107378

**Published:** 2024-05-16

**Authors:** Hana Yuasa, Naho Morino, Takumi Wagatsuma, Masayuki Munekane, Sachiko Ueda, Mayu Matsunaga, Yasuo Uchida, Takane Katayama, Toshihiko Katoh, Taiho Kambe

**Affiliations:** 1 Division of Integrated Life Science, Graduate School of Biostudies, Kyoto University, Kyoto, Japan; 2 Graduate School of Medical Sciences, Kanazawa University, Kanazawa, Japan; 3 Department of Molecular Systems Pharmaceutics, Graduate School of Biomedical and Health Sciences, Hiroshima University, Hiroshima City, Japan

**Keywords:** Zn^2+^, *N*-glycosylation, transporter, Golgi mannosidase II (GMII), lysosomal mannosidase (LAMAN), ZNT, early secretory compartments, pancreatic cancer cell growth, xenograft, quality control

## Abstract

The stepwise addition of monosaccharides to *N*-glycans attached to client proteins to generate a repertoire of mature proteins involves a concerted action of many glycosidases and glycosyltransferases. Here, we report that Golgi α-mannosidase II (GMII), a pivotal enzyme catalyzing the first step in the conversion of hybrid- to complex-type *N*-glycans, is activated by Zn^2+^ supplied by the early secretory compartment-resident ZNT5-ZNT6 heterodimers (ZNT5-6) and ZNT7 homodimers (ZNT7). Loss of ZNT5-6 and ZNT7 function results in marked accumulation of hybrid-type and complex/hybrid glycans with concomitant reduction of complex- and high-mannose-type glycans. In cells lacking the ZNT5-6 and ZNT7 functions, the GMII activity is substantially decreased. In contrast, the activity of its homolog, lysosomal mannosidase (LAMAN), is not decreased. Moreover, we show that the growth of pancreatic cancer MIA PaCa-2 cells lacking ZNT5-6 and ZNT7 is significantly decreased in a nude mouse xenograft model. Our results indicate the integral roles of ZNT5-6 and ZNT7 in *N*-glycosylation and highlight their potential as novel target proteins for cancer therapy.

Most secretory and membrane-bound proteins undergo various post-translational modifications during the secretory process. Glycosylation is a major post-translational modification, which can enhance solubility, improve folding, facilitate secretion, modulate antigenicity, and increase the *in vivo* half-life of proteins (1, 2). Consequently, glycosylation is crucial for various physiological events involving cell signaling, cell migration, cell–cell adhesion, and cell growth (3, 4, 5). Moreover, alterations in *N*-glycosylation are implicated in a wide range of pathological conditions, including cancer metastasis, chronic inflammatory diseases, and viral pathogenesis (6, 7, 8). Highly branched complex-type oligosaccharides on the cell surface are commonly associated with a malignant phenotype (9, 10, 11). Therefore, *N*-glycosylation is considered a potential target for anticancer therapeutics (11, 12).

An estimated 2% of genes in the mammalian genome encode proteins involved in the production and modification of glycans (13). Most of the glycosidases and glycosyltransferases, which reside in the lumen of the endoplasmic reticulum and Golgi apparatus (referred to as the “early secretory compartments” in this study), play essential roles in constructing the glycan repertoire on client proteins in a stepwise manner (5, 13). Many of these enzymes require metal cofactors for their activity (14, 15, 16, 17, 18). For example, ER α-mannosidase I and Golgi α-mannosidase I, belonging to the glycoside hydrolase family 47 (GH47 family), which are essential for the removal of all glucose and several mannose residues from nascent glycan chains before the extension of *N*-glycosylation, require calcium (Ca^2+^) (19, 20). The sarco/endoplasmic reticulum Ca^2+^-ATPase (SERCA) family members supply Ca^2+^ to these enzymes (18, 21). Many Golgi-resident glycosylation enzymes, such as *N*-acetylglucosaminyltransferases (MGATs) and galactosyltransferases, which are essential for the elongation and branching of *N*-glycans and, thus, for their diversity and complexity (15, 22, 23, 24), require manganese (Mn^2+^) as a common cofactor. Recently, a number of metal transport proteins have been reported to play pivotal roles in supplying Mn^2+^ to these enzymes. For example, TMEM165, Zrt, Irt-related protein 8 (ZIP8), and ZIP9 are involved in the maturation of complex-type *N*-glycans and *O*-glycans (21, 25, 26, 27, 28, 29, 30, 31). In yeast, Pmr1, a homolog of Golgi secretory pathway Ca^2+^ channel 1 (SPCA1), assays an essential function in *N*- and *O*-glycosylation (26, 32). These metal transport proteins transport Mn^2+^ in addition to their original substrate metal ions (Ca^2+^ for TMEM165 and SPCA1, and Zn^2+^ for ZIP8 and ZIP9) and are significant as Mn^2+^-supplying routes to Golgi-resident Mn^2+^-dependent glycosylation enzymes, although, the involvement of ZIP9 remains unclear.

Golgi α-mannosidase II (GMII, MAN2A1), belonging to the GH38 family of proteins, plays a critical role in the conversion of hybrid- to complex-type *N*-glycans by cleaving two different glycosidic linkages to remove two terminal mannoses (33). The *Drosophila* homolog of GMII contains Zn^2+^ at its active site (33, 34). However, how GMII acquires Zn^2+^ and to what extent is this cation important for its activity are important aspects that remain unelucidated. Considering that Zn^2+^ is coordinated at the active site of GMII, Zn^2+^ transporters must supply the cation within the Golgi for GMII activation.

In this study, we aimed to examine whether the ZNT5-6 heterodimer and the ZNT7 homodimer (hereafter, ZNT5-6 and ZNT7), which localize to the early secretory pathway (35), play an essential role in the complex-type *N*-glycosylation by supplying Zn^2+^ to GMII, thereby, contributing to the quality control of proteins. Moreover, to assess the implications of the role of ZNT5-6 and ZNT7 in *N*-glycosylation, we investigated whether Zn^2+^ starvation of GMII through impairing them could be a potential therapeutic strategy against cancer.

## Results

### *N*-glycosylation of LAMP1 and LAMP2 is impaired in HAP-*Z5Z7*-DKO cells

**Figure 1:**
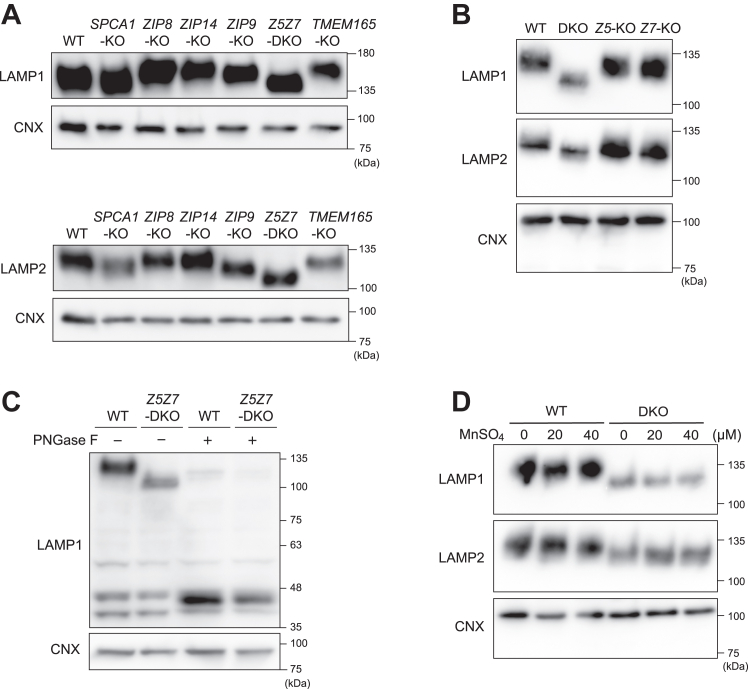
***N*-Glycosylation of LAMP1 and LAMP2 was impaired in HAP-*Z5Z7*-DKO cells.***A*, electrophoretic mobility of LAMP1 (*top panel*) and LAMP2 (*bottom panel*) was increased in HAP-*Z5Z7*-DKO cells compared with that in WT HAP1 cells and other examined HAP1 mutants. Membrane fractions prepared from the indicated cell lines were subjected to immunoblot analysis. *B*, electrophoretic mobility of LAMP1 (*top panel*) and LAMP2 (*bottom panel*) was almost unchanged in HAP-*Z5*-KO and HAP-*Z7*-KO cells. *C*, LAMP1 and LAMP2 in the membrane fractions prepared from HAP-*Z5Z7*-DKO cells had almost the same electrophoretic mobility as those in the fractions prepared from WT HAP1 cells after PNGase F digestion. (+): treated; (−): not treated. *D*, Mn^2+^ supplementation in the culture medium for HAP-*Z5Z7*-DKO cells failed to restore the electrophoretic mobility of LAMP1 and LAMP2. CNX was used as a loading control. Each experiment was performed at least three times, and representative results from independent experiments are shown.

We examined the electrophoretic mobility of extensively and highly *N*-glycosylated proteins, LAMP1 and LAMP2 (36, 37), using cell lysates prepared from wild-type (WT) HAP1 cells and HAP1 cells deficient in both *ZNT5* and *ZNT7* genes (HAP-*Z5Z7*-DKO (double-KO) cells) (38) on acrylamide gels followed by immunoblotting. We simultaneously examined the electrophoretic mobility of LAMP1 and LAMP2 in lysate of HAP1 cells lacking other metal transport proteins, namely TMEM165, ZIP8, ZIP9, SPCA1, and ZIP14 (HAP-*TMEM165*-KO, HAP-*ZIP8*-KO, HAP-*ZIP9*-KO, HAP-*SPCA1*-KO, and HAP-*ZIP14*-KO cells, respectively; Table 1). The cells lacking ZIP14 were used because this protein is homologous to ZIP8 and thus has a similar ability to transport Mn^2+^ (39, 40, 41). The electrophoretic mobility of LAMP1 and LAPM2 in lysates of HAP-*TMEM165*-KO, HAP-*ZIP8*-KO, HAP-*ZIP9*-KO, HAP-*SPCA1*-KO, and HAP-*ZIP14*-KO cells was similar or almost similar to that for the lysates of WT HAP1 cells. However, the mobility of these proteins in the lysate of HAP-*Z5Z7*-DKO cells was substantially increased (Fig. 1*A*), although the differences in electrophoretic mobility of LAMP1 and LAMP2 were not evident for HAP-*Z5*-KO and HAP-*Z7*-KO (Fig. 1*B*). The difference in the mobility for HAP-*Z5Z7*-DKO cells was attributed to impaired *N*-glycosylation because PNGase F treatment eliminated the difference in the mobility for WT HAP1 and HAP-*Z5Z7*-DKO cells (Fig. 1*C*). Mn^2+^ supplementation had no effect on the electrophoretic mobility of LAMP1 and LMAP2 (Fig. 1*D*), unlike for cells lacking TMEM165 (27, 42). These results confirmed that Mn^2+^ was not involved in the *N*-glycosylation defects found in HAP-*Z5Z7*-DKO cells. Moreover, these results suggest that Zn^2+^ supply mediated by ZNT5-6 and ZNT7 plays pivotal roles in *N*-glycosylation.

**Table 1:** Mutations introduced in *ZNT5* and *ZNT7* in the cells used in this study

Cells	Targeted region	Mutation
HAP-*ZIP9*-KO	Exon 1 in *ZIP9*	1 bp insertion in *ZIP9*
HAP-*ZIP8*-KO	Exon 5 in *ZIP8*	10 bp deletion in *ZIP8*
HAP-*ZIP14*-KO	Exon 2 in *ZIP14*	10 bp deletion in *ZIP14*
HAP-*TMEM165*-KO	Exon 1 in *TMEM165*	11 bp deletion in *TMEM165*
HAP-*SPCA1*-KO	Exon 7 in *SPCA1*	1 bp insertion in *SPCA1*
HAP-*GMII*-KO	Exon 4 in *GMII*	1 bp insertion in *GMII*
HAP-*LAMAN-*KO	Exon 2 in *LAMAN*	2 bp deletion in *LAMAN*
HAP-*Z4*-KO	Exon 2 in *ZNT4*	1 bp insertion in *ZNT4*
HAP-*Z4Z5Z7*-TKO	Exon 2 in *ZNT4*, Exon 11 in *ZNT5*, exon 2 in *ZNT7*	1 bp insertion in *ZNT4*, 2 bp deletion in *ZNT5*, 28 bp deletion in *ZNT7*
MIA-*Z5Z7*-DKO	Exon 11 in *ZNT5*, exon 2 in *ZNT7*	1 or 3 bp deletion in *ZNT5,* 1 or 58 bp deletion in *ZNT7*

Information on HAP-*Z5Z7*-DKO, HAP*-Z5*-KO, HAP*-Z7*-KO, HAP-*Z4*-KO, A549-*Z5Z7*-DKO, and SK-*Z5Z7*-DKO is presented in ref. (38, 46, 57).

### Deficiency in ZNT5-6 and ZNT7 functions impairs the complex-type *N*-glycan biosynthetic pathway

**Figure 2:**
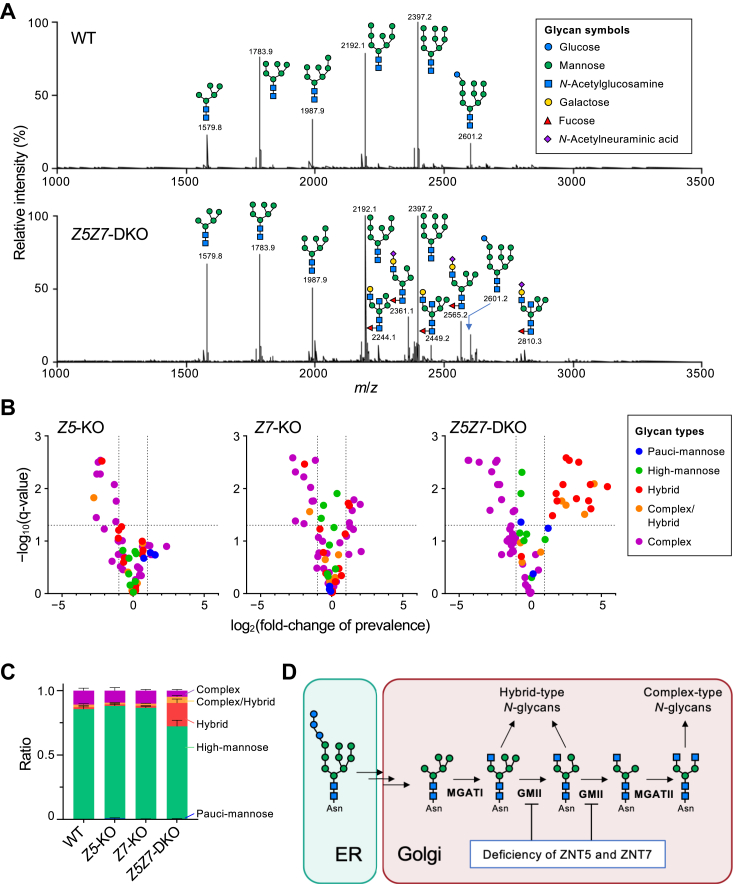
***N*-glycomics of *ZNT5*-and/or *ZNT7*-deficient HAP1 cells.***A*, full-mass profiles of permethylated *N*-glycans released by PNGase F from WT HAP1 (*upper panel*) and HAP-*Z5Z7*-DKO (*lower panel*) cells. Glycan cartoons indicate the representative deduced glycan structures corresponding to each ion peak. *B*, volcano plots for prevalence (% of the total) of each glycan ion peak for the mutant cells (left: HAP-*Z5*-KO; middle: HAP-*Z7*-KO; right: HAP-*Z5Z7*-DKO) compared with that for WT cells (n = 3). Glycan types are marked in colors. See Table S1 for details. *C*, ratios of glycan types in WT and mutant cells. *D*, deficiency of ZNT5 and ZNT7 affects the *N*-glycan synthetic pathway by inhibiting GMII. MGATI: *N*-acetylglucosaminyltransferase I; MGATII: *N*-acetylglucosaminyltransferase II.

Next, we performed an *N*-glycomic analysis to clarify the defects in *N*-glycosylation in HAP-*Z5Z7*-DKO cells. We detected 55 glycan ion peaks in the MALDI-TOF/MS spectra for WT HAP1, HAP-*Z5Z7*-DKO, HAP-*Z5*-KO, and HAP-*Z7*-KO cells (Table S1, Fig. S1). These peaks were quantified for relative abundance (percentage to the total ion intensities within the sample) and classified into the five glycan types based on their glycosyl compositions deduced from the MS/MS fragmentation analysis to confirm glycan structures when necessary. WT HAP1 cells predominantly expressed high-mannose-type *N*-glycans (prevalence: >87%), followed by complex-type (8.7%), complex/hybrid-type (3.2%), hybrid-type (1.2%), and pauci-mannose-type (0.4%) (Fig. 2, *A*, *B*, and *C*). Among the *ZNT*-deficient cells, alteration in *N*-glycome was, as expected, the most apparent in HAP-*Z5Z7*-DKO cells for which the ions peaks corresponding to the hybrid-type glycans were evident in the mass spectrum (Figs. 2*A* and S2), with a marked increase in the relative abundance of hybrid-type and complex/hybrid glycans (15% and 8.7%, respectively) and a concomitant reduction in the sum of complex- and high-mannose-type glycans (3.7% and 72%, respectively) (Fig. 2*C*). Moreover, volcano plots for fold-change in the respective glycan ion species (vs. WT HAP1) showed a robust reduction in complex-type glycans and an increase in most of the hybrid-type and complex/hybrid-type glycans, which was statistically significant for HAP-*Z5Z7*-DKO cells (Fig. 2*B*). This trend clearly indicated that deficiency of both ZNT5-6 and ZNT7 functions could impair the activity of GMII, which is a key enzyme for biosynthesis of bi- and multi-antennary complex-type *N*-glycans that trims two α1,3/6-linked mannose (Man) residues leading to the addition of the “second” nonreducing terminal *N*-acetylglucosamine (GlcNAc) residue by *N*-acetylglucosaminyltransferase II (MGATII) (Fig. 2*D*). The ratio of glycan types in HAP-*Z5*-KO and HAP-*Z7*-KO was almost identical to that for WT HAP1 (Fig. 2*C*), as evident from the electrophoretic mobility of LAMP1 and LAMP2 (Fig. 1*B*), which indicated their alternate complementarity (Fig. 2, *B* and *C*). However, the prevalence of specific glycan species was influenced by each of the selected gene deficiencies (Fig. 2*B*). Collectively, comparisons of the *N*-glycomic profiles among these HAP1 cells revealed that ZNT5-6 and ZNT7 are crucial for maintaining the homeostasis of the *N*-glycan biosynthetic pathway *via* their effect on the GMII activity.

Lysosomal α-mannosidase (LAMAN, MAN2B1) is a GMII homolog (43) in the GH38 family. It degrades α-mannosidic linkages of free *N*-glycans (FNGs), which are derived from lipid-linked oligosaccharides and the glycan donors in *N*-glycosylation, and also generated by enzymatic deglycosylation of misfolded glycoproteins during ER-associated degradation (44). We analyzed FNGs using soluble fractions prepared from WT HAP1 and HAP-*Z5Z7*-DKO cells. Contrary to the results for the *N*-glycomics of membrane fractions, no significant differences were observed among FNGs (Fig. S3). These results suggest that the LAMAN activity is not substantially impaired in HAP-*Z5Z7*-DKO cells.

### GMII activity is substantially decreased in *Z5Z7*-DKO cells

**Figure 3:**
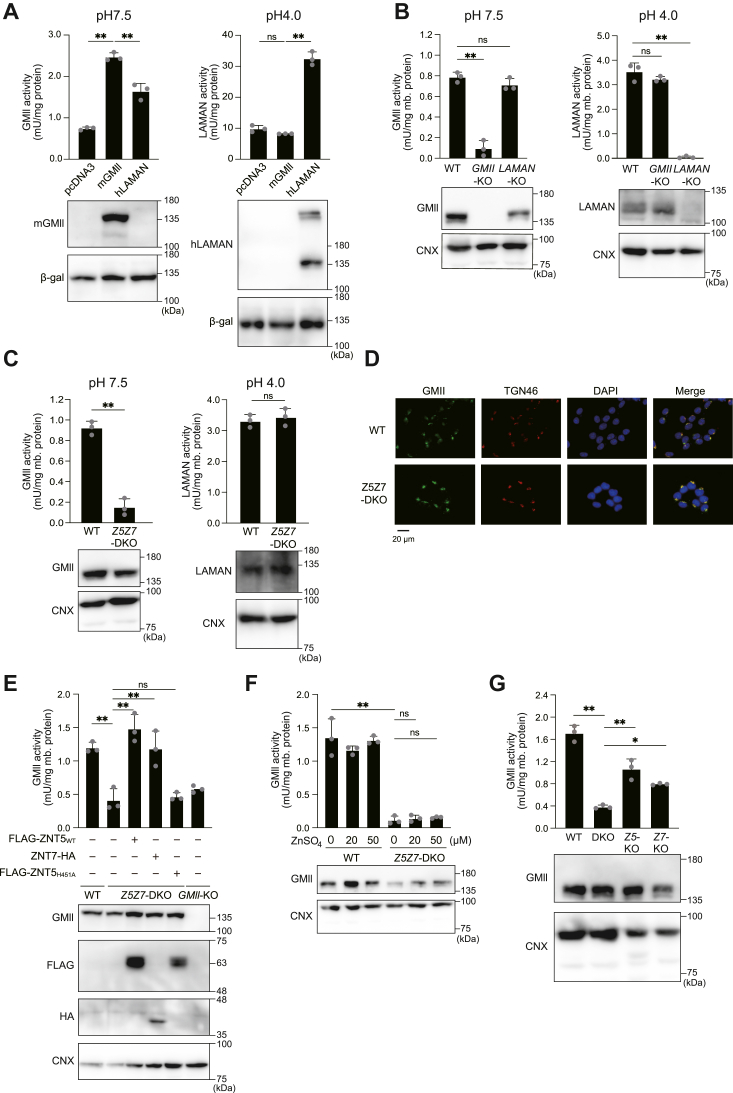
**GMII activity was substantially decreased in HAP-*Z5Z7*-DKO cells, although its expression was almost unchanged.***A and B*, the activity of GMII was distinguished from that of LAMAN by changing the pH of the reaction buffer. In *A*, the activity was measured using the total cell lysates prepared from SK-MEL-2 cells transiently transfected with mouse GMII (mGMII) or LAMAN expression plasmid. In *B*, the accurate measurement of GMII activity was confirmed using the membrane fractions prepared from HAP-*GMII*-KO or HAP-*LAMAN*-KO cells. *C*, GMII activity was substantially decreased in HAP-*Z5Z7*-DKO cells (*left*), whereas LAMAN activity was not (*right*). *D*, in HAP-*Z5Z7*-DKO cells, GMII localized with the Golgi apparatus. TGN46 was used as the Golgi marker. E. In HAP-*Z5Z7*-DKO cells, GMII activity was reversed by co-expression of ZNT5 or ZNT7, but not by Zn^2+^-transport incompetent ZNT5 mutant (ZNT5_H451A_). *F*, the reduced GMII activity in HAP-*Z5Z7*-DKO cells was not restored upon Zn^2+^ supplementation. G. GMII activity was partially decreased in HAP-*Z5*-KO or HAP-*Z7*-KO cells. GMII activity is expressed as mean ± SD of values from triplicate experiments. mb. protein: membrane protein; ∗∗: *p* < 0.01; ∗: *p* < 0.05; ns: not significant. Each experiment was performed at least thrice, and representative results from independent experiments are presented.

We investigated whether the GMII activity was impaired in *Z5Z7*-DKO cells. For this, we established a simple method to distinctly measure the activity of GMII and LAMAN using *p*-nitrophenyl α-d-mannopyranoside as a substrate. We first prepared total cellular lysates from SK-MEL-2 cells, transiently transfected with mouse GMII (mGMII) or LAMAN expression plasmids (SK-MEL-2 cells were used considering their high transfection efficiency (45)), and used them to measure the respective enzyme activity by changing the pH of the reaction buffer. The activity of mGMII could be measured at pH 7.5, whereas that of LAMAN was detected only at a low pH of 4.0 (Fig. 3*A*). We then confirmed if the activities of these enzymes could be discriminated. We found that the respective mannosidase activity was substantially decreased in the membrane protein fraction prepared from HAP1 cells deficient in *GMII/MAN2A1* (HAP-*GMII*-KO cells) or *LAMAN/MAN2B1* (HAP-*LAMAN*-KO cells). Specifically, the GMII activity was only decreased in the membrane fraction of HAP-*GMII*-KO cells but not in that of HAP-*LAMAN*-KO, at pH 7.5 (Fig. 3*B*, *left)*, whereas the LAMAN activity was substantially decreased for HAP-*LAMAN*-KO cells but not for HAP-*GMII*-KO cells, at pH 4.0 (Fig. 3*B*, *right*). These results indicate that the GMII activity could be distinctly measured using our system.

The GMII activity in HAP-*Z5Z7*-DKO cells was substantially lower than that in WT HAP1 cells (Fig. 3*C*, *left*). However, the protein levels of GMII were comparable between the two cell types (Fig. 3*C*, *left*), and the Golgi localization of the proteins was not impaired in HAP-*Z5Z7*-DKO cells (Fig. 3*D*), suggesting that GMII is present as an *apo*-enzyme in HAP-*Z5Z7*-DKO cells. This feature is different from tissue non-specific alkaline phosphatase (TNAP), which is promptly degraded through both the lysosomal and proteasomal degradation pathways in HAP-*Z5Z7*-DKO cells (38). In contrast, the LAMAN activity in HAP-*Z5Z7*-DKO cells was comparable to that in WT HAP1 cells (Fig. 3*C*, *right*), consistent with the results of the FNG analysis (Fig. S3). The substantial reduction in the GMII activity in HAP-*Z5Z7*-DKO cells, which was almost the same as that in HAP-*GMII*-KO cells, was restored by re-expression of ZNT5 or ZNT7, but not by that of Zn^2+^-transport incompetent ZNT5 (ZNT5_H451A_) (Fig. 3*E*). Supplementation of Zn^2+^ also failed to restore the GMII activity (Fig. 3*F*). The GMII activity was partially decreased in HAP-*Z5*-KO and HAP-*Z7*-KO cells (Fig. 3*G*), as indicated by the reduction in several complex-type glycans in the single KO cells in the *N*-glycomics analysis (Fig. 2*B*).

**Figure 4:**
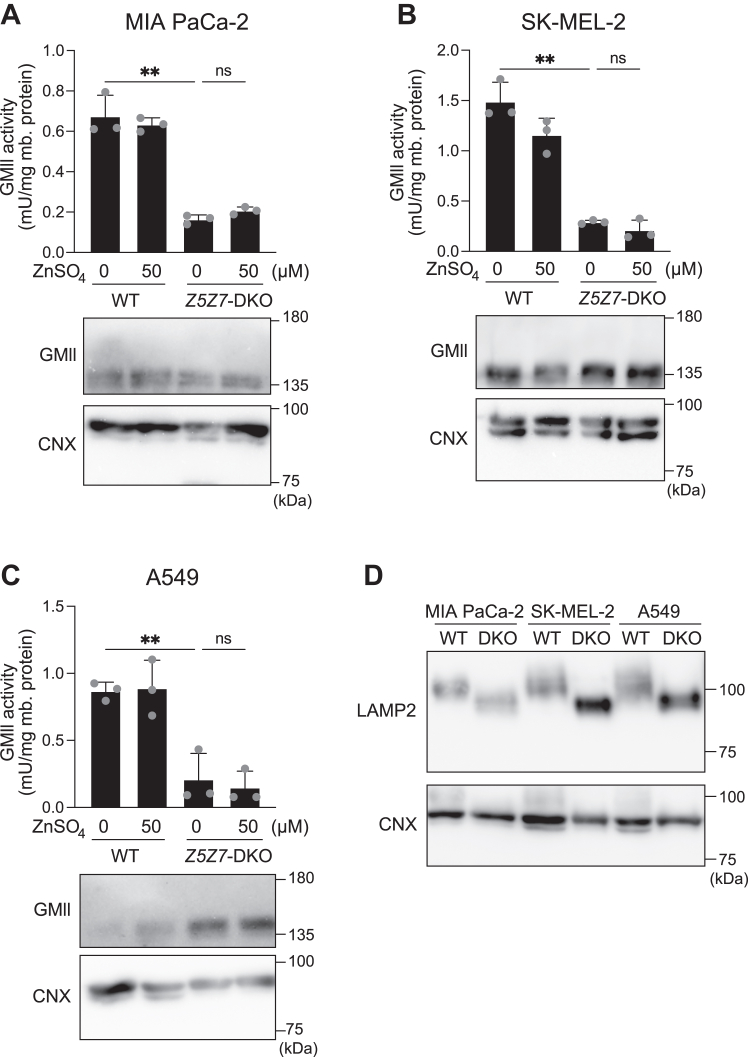
**Essentiality of Zn**^**2+**^**supply by ZNT5-6 and ZNT7 to GMII is conserved in other cultured human cells.***A–C*, GMII activity was substantially decreased in *Z5Z7*-DKO cells compared with that in WT cells, and it was not restored upon Zn^2+^ supplementation. MIA PaCa-2 (*A*), SK-MEL-2 (*B*), and A549 (*C*) cells were examined. GMII activity is expressed as mean ± SD of values from triplicate experiments. mb. protein: membrane protein; ∗∗: *p* < 0.01; ns: not significant. *D*, Electrophoretic mobility of LAMP2 was increased in MIA-*Z5Z7*-DKO, SK-*Z5Z7*-DKO, and A549-*Z5Z7*-DKO cells compared with that in their respective WT cells. Each experiment was performed at least thrice, and representative results from independent experiments are presented.

Next, we confirmed that the loss of ZNT5-6 and ZNT7 functions resulted in the enhanced electrophoretic mobility of LAMP1 and decreased GMII activity in other types of human cells, including MIA PaCa-2, SK-MEL-2, and A549 cells deficient in *ZNT5* and *ZNT7* (MIA-*Z5Z7*-DKO (Table 1), SK-*Z5Z7*-DKO, and A549-*Z5Z7*-DKO (46)) (Fig. 4, *A*–*D*). These results indicate that ZNT5-6 and ZNT7 play a pivotal role in the activation of GMII by supplying Zn^2+^ in the early secretory compartments.

### Glycoprotein expression is decreased in *Z5Z7*-DKO cells

To examine whether the expression profile of *N*-glycosylated proteins was altered in HAP-*Z5Z7*-DKO cells, we performed gene ontology (GO) term enrichment analysis using the top 50 proteins that showed differences in their relative expression between WT HAP1 and HAP-*Z5Z7*-DKO cells, determined using quantitative proteomics with sequential window acquisition of all theoretical fragment ion spectra mass spectrometry (SWATH-MS) analysis of membrane fractions prepared from both the cell lines (ProteomeXchange Consortium with the dataset identifier PXD032172 (46)). “Glycoprotein” was the third most-enriched GO term, whose expression decreased in HAP-*Z5Z7*-DKO cells (Table 2). We also performed the GO analysis using the top 50 proteins by identifying 2864 proteins between WT MIA PaCa-2 and MIA-*Z5Z7*-DKO cells (the dataset identifier PXD032172, Table S2); the most enriched protein term whose expression decreased in MIA-*Z5Z7*-DKO cells was “Glycoprotein” (Table 3). These results suggest that the expression of proteins that are *N*-glycosylated is profoundly affected by the loss of ZNT5-6 and ZNT7 functions, and clearly show that ZNT5-6 and ZNT7 play crucial roles in protein quality control through the *N*-glycosylation pathway *via* GMII activation.

**Table 2:** Functional annotation clustering (post-translational modification) showing decreased protein expression in HAP-*Z5Z7*-DKO cells compared with that in WT HAP1 cells using the top 50 proteins in the SWATH-MS analysis

Term	Number within the top 50 proteins	Fold enrichment	Raw *p-*value	FDR
GPI-anchor	5	11.12	9.52E-04	1.33E-02
Lipoprotein	6	2.04	1.57E-01	3.00E-01
Glycoprotein	22	1.46	3.55E-02	1.24E-01
Disulfide bond	17	1.40	1.10E-01	2.56E-01

Raw data from the SWATH-MS analysis are shown in ref. (46). Fifty proteins with the smallest DKO/WT ratios for protein expression levels were subjected to GO enrichment analysis (cellular component). Four enriched components are listed in the table. The number within the top 50 proteins, fold enrichment, raw *p* value, and FDR were obtained *via* the original algorithm on the DAVID website.

FDR, false discovery rate; GO, Gene ontology.

**Table 3:** Functional annotation clustering (post-translational modification) showing decreased protein expression in MIA-*Z5Z7*-DKO cells compared with that in WT MIA PaCa-2 cells using the top 50 proteins in the SWATH-MS analysis

Term	Number within the top 50 proteins	Fold enrichment	Raw *p*-value	FDR
Glycoprotein	25	1.55	9.68E-03	6.90E-02
Disulfide bond	18	1.39	1.06E-01	2.91E-01

Raw data from the SWATH-MS analysis are shown in Table S3. GO enrichment analysis was performed as described in Table 2.

### Effects of the loss of ZNT5-6 and ZNT7 functions on the growth of pancreatic cancer cells in a nude mouse xenograft model

**Figure 5:**
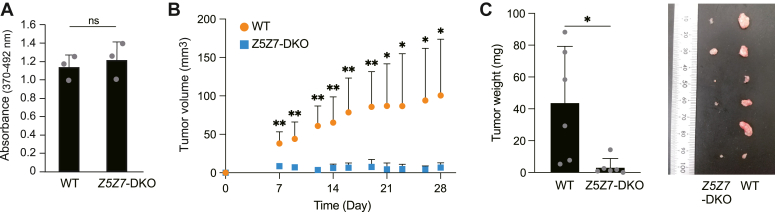
**Loss of ZNT5-6 and ZNT7 functions impaired the pancreatic cancer cell growth in the nude mouse xenograft model.***A*, cell proliferation was not significantly different between MIA PaCa-2 and MIA-*Z5Z7*-DKO cells. Cell proliferation was determined by measuring BrdU incorporation as described in Experimental procedures (*n* = 3). ns: not significant. *B*, volumes of tumors derived from *s.c.*-inoculated MIA-*Z5Z7*-DKO cells were significantly decreased compared with those of tumors derived from WT MIA PaCa-2 cells. Tumor volumes were measured up to 4 weeks (*n* = 6). ∗∗: *p* < 0.01; ∗: *p* < 0.05. *C*, weights of tumors removed from nude mice were significantly decreased in MIA-*Z5Z7*-DKO cells. Pictures of each tumor from each group are shown. ∗: *p* < 0.05. Each experiment was performed at least thrice, and representative results from independent experiments are presented.

As highly branched complex-type oligosaccharides are commonly associated with a malignant phenotype (9, 10, 11), GMII has received attention as an attractive therapeutic target for preventing cancer progression (11, 47). We examined the possibility that functional impairment of ZNT5-6 and ZNT7 may affect the characteristics of malignant cancer cells, MIA PaCa-2, in an immunodeficient nude mouse xenograft model (48). After confirming that the growth of MIA-*Z5Z7*-DKO cells in culture was almost the same as that of WT MIA PaCa-2 cells (Fig. 5*A*), these cells were subcutaneously (*s.c*.)-inoculated into the right or left shoulder areas of nude mice. The MIA-*Z5Z7*-DKO cells showed significantly reduced growth for 4 weeks in xenograft compared with the WT cells (Figs. 5*B* and S4). Consistent with this, weights of tumors removed after 4 weeks from mice *s.c*.-injected with MIA-*Z5Z7*-DKO cells were significantly decreased compared with those from mice injected with WT MIA PaCa-2 cells (Fig. 5*C*). These results indicate that functional impairment of ZNT5-6 and ZNT7 contributes to the decrease in pancreatic cancer cell progression *in vivo*.

## Discussion

We show that the activation of GMII in the early secretory compartments, which is essential for *N*-glycosylation, is mediated by Zn^2+^ supplied by ZNT5-6 and ZNT7. Loss of ZNT5-6 and ZNT7 functions increased the sum of hybrid-type and complex/hybrid glycans and concomitantly reduced the sum of complex- and high-mannose-type glycans, which led to the altered expression pattern of *N*-glycosylated proteins. We also show that loss of ZNT5-6 and ZNT7 functions may contribute to reduced malignancy of MIA PaCa-2 cells probably through reduced complex-type *N*-glycosylation. The evidence clearly indicates the novel cellular functions of Zn^2+^ through ZNT5-6 and ZNT7 in the early secretory compartments and provides novel insights into the mechanism of regulation of glycosylation by a trace element. Our results should potentially be useful in devising a novel strategy for cancer therapy.

The present study was prompted by our finding of a profound increase in the electrophoretic mobility of LAMP1 and LAMP2 by changes in *N*-glycosylation during the course of the study. Similar defects were found in our previous study wherein we noted that the electrophoretic mobility of the highly *N*-glycosylated angiotensin-converting enzyme 2 was significantly enhanced in *Z5Z7*-DKO cells compared with that in WT cells (46). In addition to these highly *N*-glycosylated proteins, alterations in the global expression of *N*-glycosylated proteins were noted in *Z5Z7*-DKO cells compared with that in WT cells. No severe growth defects were noticed in *Z5Z7*-DKO cells under usual culture conditions, which may be because of the several homeostatic responses in *Z5Z7*-DKO cells. The increased expression of MGATI, MGATII and β-1,4-galactosyltransferase 1 (B4GALT1) in HAP-*Z5Z7*-DKO and MIA-*Z5Z7*-DKO cells (B4GALT7 was also detected) in the SWATH-MS analysis (Tables S3 and S4) also gives credence to this notion. These responses may be important as the “Golgi stress responses” described recently (49, 50). Further investigation is required, considering that more than one-third of synthesized proteins encoded by the human genome traverse to the secretory pathway (51).

The terms “GPI-anchor,” “Disulfide bond,” and “Lipoprotein” were enriched in the SWATH-MS analysis in addition to “Glycoprotein.” We previously reported that GPI-anchored biosynthesis was impaired in *Z5Z7*-KO cells, probably because of the loss of functions of three PIG proteins (PIG-N, PIG-O, and PIG-G) (46). Moreover, the functions of protein disulfide isomerase (PDI) family protein are controlled by Zn^2+^ (52, 53, 54) and thus the enrichment of the term “Disulfide bond” was not notable if Zn^2+^, mediated by ZNT5-6 and ZNT7, is important for regulating PDI functions. In contrast, the relationship between Zn^2+^ and lipoprotein is less clear. Thus, the enrichment of the term “Lipoprotein” is notable from the view of zinc functions in the early secretory pathway, which should be examined in future studies. These results indicate that ZNT5-6 and ZNT7 play pivotal roles in the post-translational modification of proteins through transporting Zn^2+^ into the early secretory pathway in addition to the “Golgi stress responses,” as clarified in this study and described above.

Alterations in *N*-glycomic profiles were most apparent in HAP-*Z5Z7*-DKO cells, and the profiles in HAP-*Z5*-KO and HAP-*Z7*-KO were almost identical to those in WT HAP1 cells (Fig. 2*C*). However, the prevalence of the specific glycan species was affected in HAP-*Z5*-KO and HAP-*Z7*-KO cells (Fig. 2*B*). These differences indicate that ZNT5-6 and ZNT7 do not necessarily have the same function despite the similarity in their Zn^2+^ transporting activity and subcellular localization in the early secretory pathway. A recent report revealed that ZNT5-6 and ZNT7 show different subcellular localization, with the former mainly localized in the *medial*-Golgi and the latter in the *cis* Golgi (54). Moreover, ZNT5-6 and ZNT7 show obvious physiopathological differences in human and medaka fish (*Oryzias latipes*). In humans, mutations in *SLC30A5/ZNT5* or *SLC30A7/ZNT7* resulted in perinatal lethal cardiomyopathy or stunted growth, testicular hypoplasia, and bone marrow failure, respectively (55, 56). *Znt5*^+/−^;*Znt7*^−/−^ medaka respond to touch, but not *Znt5*^−^^/^^−^;*Znt7*^+/^^−^ (46). Considering these facts, GMII activity may be regulated in a different manner by ZNT5-6 and ZNT7, for example, in a cell-type-specific or stimulant-dependent manner, although substantial defects are obvious only in *Z5Z7*-DKO cells in a cell culture model. Elucidation of specific physiopathological functions of ZNT5-6 and ZNT7 would facilitate our increased understanding of the homeostatic control of the secretory pathway.

Although Zn^2+^ supplied by ZNT5-6 and ZNT7 is required for *N*-glycosylation through GMII, it is apparently not required for the degradation of α-mannosidic linkages of FNGs through LAMAN (see Fig. S3, *A* and *B*). These results were reflected in the activities of GMII and LAMAN in HAP-*Z5Z7*-DKO cells. The GMII activity was dependent on ZNT5-6 and ZNT7, whereas that of LAMAN showed no such dependence. It is important to identify the reason for this discrepancy. One reason could be the difference in the subcellular localization. GMII localizes to the Golgi, whereas LAMAN is present in lysosomes. We compared the amounts of FNG species in HAP1 cells deficient in *ZNT4* (HAP-*Z4* cells), which localizes to the lysosomes (57), and HAP1 cells deficient in *ZNT5*, *ZNT7*, and *ZNT4* (HAP-*Z4Z5Z7*-TKO cells). The catabolism of FNGs was not much affected by the loss of ZNT4 in the respective cells compared with that in WT or HAP-*Z5Z7*-DKO cells (Fig. S3, *B*–*D*). The possibility that only lysosomal enzymes acquire Zn^2+^ through their unique pathways was excluded because the activation of the lysosomal enzyme sphingomyelin phosphodiesterase 1 (SMPD1) is completely dependent on Zn^2+^ mediated through ZNT5-6 and ZNT7 in the early secretory pathway. At present, we are unable to explain how these proteins receive Zn^2+^ in a specific manner. Nonetheless, clarification of this point would provide insights into how Zn^2+^ metalation of the intracellular Zn^2+^-enzymes is sophisticatedly controlled.

Altered glycosylation in cancer cells allows new interactions with immune cells to suppress antitumor immunity by evading immune surveillance (58, 59). GMII suppresses the sensitivity of cancer cells to T-cell-mediated death (60), and inhibitors of the GMII activity have been extensively explored as potential cancer therapeutics. Swainsonine, a potent inhibitor of this protein, and its related compounds improve the outcome in breast, colon, and skin cancers (12, 61), and have been administrated in clinical trials (11, 12, 62). However, these inhibitors have not been clinically applied in view of their side effects, which resemble α-mannosidosis, a lysosomal storage disease, resulting from the off-target inhibition of LAMAN (11, 63, 64). Our xenograft model showed that the loss of ZNT5-6 and ZNT7 functions impaired pancreatic cancer cell growth, likely due to the altered *N*-glycosylation through a substantial decrease in the GMII activity, although other cellular pathways may be affected. Notably, the loss of ZNT5-6 and ZNT7 functions had minor effects on the degradation of α-mannosidic linkages of FNGs by LAMAN. This specificity offers an advantage for GMII inhibition, and thus ZNT5-6 and ZNT7 may be novel chemotherapeutic target proteins.

Another important aspect of this study is the demonstration of a method for distinguishing between the GMII and LAMAN activities using *p*-nitrophenyl α-d-mannopyranoside simply by changing the pH of the reaction buffer. This simple method employing membrane fractions, with the removal of the contaminating activity of the cytosolic α-mannosidase (encoded by *MAN2C1*) belonging to the GH38 family (65, 66), is expected to be widely used in biochemical analyses of GMII and LAMAN in the future.

Many glycosyltransferases need Mn^2+^ as an essential divalent cation (15, 16, 17, 24), whereas several glycosidases require Ca^2+^ (19, 20). This study revealed that GMII requires Zn^2+^ for its activity. The evidence from this study indicates that the interplay and homeostatic control of these divalent cations through transport proteins are essential for constructing the *N*-glycan repertoire on the client proteins and, thereby, for the quality control of *N*-glycosylated proteins. Thus, the maintenance of metal homeostasis in the early secretory pathway compartments should be the main area of focus.

## Experimental procedures

### Cell culture

HAP1 cells, which are human near-haploid cells (Horizon Discovery), were maintained at 37 °C in a humidified 5% CO_2_ incubator in Iscove’s modified Dulbecco’s medium (Nacalai Tesque) containing 10% heat-inactivated fetal calf serum (FCS; Biosera), 100 U/ml penicillin, and 100 μg/ml streptomycin (Nacalai Tesque), as described previously (38). For Zn^2+^ deficiency experiments, Zn^2+^-deficient culture medium prepared using FCS treated with Chelex-100 resin (CX; Bio-Rad Laboratories), was used as described previously (67). For Zn^2+^ supplementation experiments, cell culture medium supplemented with various concentrations of ZnSO_4_ was used. Dulbecco’s modified Eagle medium (FUJIFILM Wako Pure Chemical) or RPMI1640 (FUJIFILM Wako Pure Chemical) was used to maintain pancreatic cancer MIA PaCa-2 (68) and lung cancer A549 (46) cells and melanoma SK-MEL-2 cells (JCRB1393, JCRB cell bank).

### Plasmid construction

The plasmids used for the expression of N-terminal FLAG-tagged human ZNT5 (FLAG-ZNT5), C-terminal HA-tagged ZNT7 (ZNT7-HA), and Zn^2+^ transport-incompetent ZNT5 mutant (FLAG-ZNT5_H451A_) were described previously (38, 69). The plasmid used for expressing GFP-fused mouse GMII (pEF-ManII-GFP, plasmid #160905, a gift from Benjamin Glick) (70) or human LAMAN (pHAGE-MAN2B1, plasmid #116756, a gift from Gordon Mills & Kenneth Scott) (71) was purchased from Addgene.

### Disruption of ZNT5 and ZNT7 or ZIP8, ZIP9, ZIP14, TMEM165, SPCA1, GMII, and LAMAN

*ZNT5* and *ZNT7* were simultaneously disrupted using CRISPR/Cas9-mediated genome editing with sgRNA expression plasmids as described previously (38). The constructed plasmids (4 μg) and one-10th quantity of pcDNA6/TR (carrying the blasticidin S resistance gene) were co-transfected into 80% confluent MIA PaCa-2 cells using 4 μl Lipofectamine 2000 (Thermo Fisher Scientific). After culturing for 1 day, the cells were transferred to a 10-cm cell culture dish and cultured in the presence of 5 μg/ml blasticidin S (InvivoGen, San Diego, CA) to establish MIA-*Z5Z7*-DKO cells clones. HAP-*Z5Z7*-DKO, SK-*Z5Z7*-DKO, and A549-*Z5Z7*-DKO cells were established in previous studies (38, 46). HAP1 cells deficient in *ZIP8*, *ZIP9*, *ZIP14*, *TMEM165*, *SPCA1*, *GMII/MAN2A1*, or *LAMAN/MAN2B1* were established in the same manner. We used 20 μg/ml blasticidin S (InvivoGen) to select the HAP1 KO cells. Oligonucleotides used for the generation of sgRNA expression plasmids are listed in Table S5. Gene editing was confirmed by sequencing the PCR fragments amplified from genomic DNA using the primers listed in Table S6.

### Transient and stable transfection

Transient transfection was performed as described previously (46). SK-MEL-2 cells were seeded in 12-well plates (1.0 × 10^5^ cells/well) and cultured for 48 h. The cells were then transfected with 1 μg of empty pcDNA3 or pcDNA3 harboring each cDNA with 0.2 μg of pβactβgal plasmid (72) in Opti-MEM (Thermo Fisher Scientific) for normalization of transfection efficiency, using Lipofectamine 2000 (Thermo Fisher Scientific). The transfection medium was replaced after 4 h with the corresponding culture medium, and the cells were cultured for an additional 24 h prior to the experiments. Stable transformants of HAP-*Z5Z7*-DKO cells were established in previous studies (38, 57).

### *N*-glycomics

Confluent cells from three 10 cm culture plates (*n* = 3) were harvested by scraping with a cell-scraper, washed twice with cold phosphate-buffered saline (PBS; 137 mM NaCl, 2.68 mM KCl, 1.47 mM KH_2_PO_4_, 8.1 mM NaH_2_PO_4_, pH 7.4), and stored at −80 °C until use. The cells were homogenized with a Dounce-type homogenizer (WHEATON) in chloroform/methanol/water (4:8:3) on ice, and proteins were pelleted by centrifugation in pointed glass tubes with screw caps. The supernatants were collected and subjected to FNG analysis as described in the following section. Protein pellets were further extracted in ice-cold acetone/water (4:1) twice to remove the remaining soluble lipids and saccharides. Proteins were then extracted in acetone, pelleted again by centrifugation, and dried under a nitrogen stream. Next, the proteins were resuspended in 200 μl of trypsin buffer [100 mM Tris-HCl (pH 8.2), 10 mM CaCl_2_] and heated at 95 °C for 5 min. After cooling to room temperature (25–30 °C), trypsin (Sigma-Aldrich, St Louis, MO) and chymotrypsin (Sigma-Aldrich) were added at final concentrations of 0.2 mg/ml, and the samples were incubated overnight at 37 °C. The tryptic/chymotryptic digestions were stopped by boiling the solution for 5 min, and the tubes were centrifuged. The supernatants were then evaporated to dryness in a centrifugal evaporator and reconstituted in 5% acetic acid. The peptides were applied onto a SepPak C_18_ cartridge column (100 mg, Waters, Milford, MA) pre-equilibrated with 5% acetic acid, followed by sequential elution with 20% 2-propanol/5% acetic acid and 40% 2-propanol/5% acetic acid. The eluates were evaporated to dryness in a centrifugal evaporator. *N*-Glycans were released from the glycopeptides by treatment with three units of PNGase F (Roche) and purified on SepPak C_18_ cartridge columns in the flow-through fractions. *N*-Glycans were further purified using a graphitized carbon column (InertSep GC column 150 mg/3 ml, GL Science, Tokyo, Japan) as described previously (73). Subsequent permethylation of purified *N*-glycans was carried out according to the standard method (74). The permethylated *N*-glycans were analyzed using matrix-associated laser desorption ionization-time of flight mass spectrometry (MALDI-TOF/MS) on an Autoflex III system (Bruker Daltonics, Billerica, MA) with 2,5-dihydroxybenzoic acid as a matrix in the positive ion and reflector modes. The MS/MS analysis of selected ion peaks was performed using the LIFT function. Shot counts and laser intensity were kept constant for comparison among the samples. Glycosyl compositions were estimated based on mass-to-charge (*m*/*z*) values of the ion peaks using the ExPASy GlycoMod tool (75). Glycan types were classified into complex, hybrid, high-mannose, and pauci-mannose types based on the glycosyl compositions deduced from the MS and MS/MS fragmentation analyses, when applicable. The term "Complex/Hybrid" indicates possible mixtures of both structures.

### Analysis of free *N*-glycans

FNGs were extracted from the supernatant obtained from samples after the pelleting of proteins, as mentioned above. Prior to extraction, 500 pmol of lacto-*N*-fucopentaose I (LNFPI) was added to each supernatant sample as an external standard oligosaccharide. The supernatant samples were dried under nitrogen with heating at 40 °C and dissolved in 1 ml water. The samples were applied onto a SepPak C_18_ cartridge column (100 mg), which was activated with acetonitrile and pre-equilibrated with water, followed by washing with 3 ml water. The flow-through and wash fractions were combined and lyophilized. FNG samples were further purified using a graphitized carbon column, lyophilized, and permethylated, as described above. Permethylated FNGs were subjected to MALDI-TOF/MS and MS/MS analyses. Semiquantification was performed using LNFPI as a standard. Glycan amounts were normalized against protein amounts assayed with the trypsin/chymotrypsin-treated peptide solutions of the *N*-glycomics samples using the BCA protein assay kit (Thermo Scientific).

### Preparation of membrane fractions

Cells were resuspended in 1 ml of cold homogenizing buffer and homogenized with 90 strokes of a 7 ml Dounce homogenizer (WHEATON). For removing the nucleus, the homogenate was centrifuged at 2300*g* for 5 min. The post-nuclear supernatant was centrifuged at 20,400*g* for 30 min at 4 °C. After washing with cold PBS, the pellet was lysed in a lysis buffer (10 mM Tris-HCl pH 7.5, 0.5 mM MgCl_2_, 0.1% Triton-X 100) and stocked as the membrane fraction at −80 °C until use.

### Immunoblotting

Immunoblotting was performed using total cellular lysates or membrane proteins (15 or 20 μg) as described previously (46, 76). The proteins were blotted onto polyvinylidene fluoride (PVDF) membranes (Millipore Corp), which were then blocked for 1 h with 5% skim milk and 0.1% Tween 20 in PBS or for 30 min with SuperBlock Blocking Buffer (Thermo Scientific) and 0.1% Tween 20 in PBS, and subsequently incubated with one of the primary antibodies (diluted in the blocking solution): anti-LAMP1 [H4A3] (1:3000; Developmental Studies Hybridoma Bank (DSHB) deposited by August, J.T./Hildreth, J.E.K.); anti-LAMP2 [H4B4] (1:3000; DSHB deposited by August, J.T./Hildreth, J.E.K.); anti-GMII [D-5] (1:3000, Santa Cruz Biochemistry); anti-GMII [F-10] (1:3000, Santa Cruz Biochemistry); anti-LAMAN [NBP3-05138] (1:1000; Novus Biologicals); anti-calnexin (CNX) [M178-3] (1:3000; MBL, Nagoya, Japan); anti-calnexin [10427-2-AP] (1:10000; Proteintech Group Inc., Chicago, IL); anti-FLAG M2 [F3165] (1:3000; Sigma-Aldrich); anti-HA [HA-11] (1:3000; BioLegend); anti-HA [561] (1:3000, MBL); or anti-β-galactosidase [6F4] (1:3000; MBL). For the detection of LAMP1 digested with PNGase F, the anti-LAMP1 [L1418] antibody (1:3000; Sigma-Aldrich) was used. Immunoreactive bands were detected using 1:3000 diluted horseradish peroxidase-conjugated anti-mouse, anti-rabbit, or anti-rat secondary antibodies (NA931, NA934, or NA935, Cytiva) and Immobilon Western Chemiluminescent HRP substrates (Millipore) or SuperSignal West Femto Maximum Sensitivity substrate (Thermo Fisher Scientific). Chemiluminescence images were obtained using ImageQuant LAS 500 (Cytiva). For removal of *N*-glycosyl groups, the membrane fractions (20 μg) were denatured in 1× Glycoprotein Denaturing Buffer (0.5% SDS, 40 mM DTT) containing 0.2% Protease Inhibitors (Nacalai Tesque) at 37 °C for 30 min. Nonidet P-40 and sodium phosphate were then added to final concentrations of 1% and 50 mM, respectively. After the addition of PNGase F (New England Biolabs), the mixture was incubated at 37 °C for 1 h. The samples were then subjected to immunoblotting.

### Immunofluorescence staining

Cells were cultured on coverslips and fixed with methanol at −20 °C for detecting GMII. These cells were incubated with anti-Mannosidase II [AB3712] (1:1000; Sigma-Aldrich) and anti-TGN46 [AHP500GT] (1:1000; Bio-Rad) as primary antibodies, and then with Alexa 488–conjugated donkey anti-rabbit IgG or Alexa594–conjugated donkey anti-goat IgG (Thermo Fisher Scientific) as the secondary antibodies, diluted in 2% BSA. The antibodies were applied for 1 h at room temperature (25–30 °C) or at 4 °C overnight, and 5 μg/ml 4,6-diamino-2-phenylindole (DAPI) (Thermo Fisher Scientific) was added during incubation with the secondary antibody to label nuclei. After washing with PBS three times, the coverslips were mounted onto glass slides using SlowFade Diamond Antifade Mountant reagent (Thermo Fisher Scientific). The stained cells were examined using a fluorescence microscope (FSX100; Olympus). Identical exposure settings and times were used while acquiring the images.

### Measurement of mannosidase activity

For measuring the GMII activity, total cell lysates or membrane proteins (30 μg) prepared from cells were mixed with a 300 μl substrate solution [3 mg/ml *p*-nitrophenyl α-d-mannopyranoside (Tokyo Chemical Industry) prepared in 10 mM Tris-HCl pH 7.5, 0.5 mM MgCl_2_, and 0.1% Triton-X100]. After incubation at 37 °C for 24 h, the released *p*-nitrophenolate was quantified by measuring the absorbance at 405 nm using a Synergy H1 Hybrid multi-mode microplate reader (BioTek). *α*-Mannosidase purified from *Canavalia ensiformis* (Sigma-Aldrich) was used to generate a calibration curve. For measuring the LAMAN activity, another substrate solution [3 mg/ml *p*-nitrophenyl α-D-mannopyranoside (Tokyo Chemical Industry) prepared in 100 mM CH_3_COOH pH 4.0] was used. After incubation at 37 °C for 24 h, 600 μl stop solution (1 M Na_2_CO_3_) was added.

### Cell proliferation assay

Cell proliferation was measured using the Cell Proliferation ELISA BrdU assay (Roche Diagnostic, Mannheim, Germany), according to the manufacturer's instructions. In brief, cells (5.0 × 10^3^) were seeded in 96-well plates and cultured for 50 h, with the addition of BrdU labeling solution during the last 2 h of incubation. Thereafter, the culture medium was removed, and the cells were fixed in FixDenat solution and incubated with an anti-BrdU-POD solution for 90 min. After washing three times, 100 μl substrate solution containing tetramethylbenzidine was added and the cells were incubated for 10 min. Absorbance at 370 and 492 nm was measured according to the manufacturer's protocol.

### Xenograft experiment

Experiments with animals were conducted in accordance with the Guidelines for the Care and Use of Laboratory Animals of Kanazawa University. The experimental protocols were approved by the Committee on Animal Experimentation of Kanazawa University. Female BALB/c nude mice were obtained from Japan SLC, Inc. The animals were housed at 23 °C under a 12-h alternating light/dark schedule and provided free access to food and water. WT MIA PaCa-2 and MIA-*Z5Z7*-DKO cells were *s.c.* injected into the right and left shoulders of 4-week-old mice, respectively. Tumor volume and body weight of mice were measured three times weekly. Tumor volumes were calculated using the formula (*x* × *y*^2^)/2, where *x* and *y* are the length and width of the tumor, respectively.

### Statistical analyses

Statistical analyses were performed using GraphPad Prism 9 (GraphPad Software; https://www.graphpad.com/). All data are expressed as mean ± standard deviation (SD) of values from triplicate experiments. Statistical significance was determined using one-way ANOVA followed by Tukey’s *post hoc* test (comparison of three or more groups) or Student’s *t* test (comparison between two groups) at *p* < 0.05 and *p* < 0.01.

## Data availability

All data generated or analyzed during this study are included in this published article and its supporting information file or are available from the corresponding author (Taiho Kambe, 10.13039/501100005683Kyoto University, E-mail: kambe.taiho.7z@kyoto-u.ac.jp) upon reasonable request. Full-length immunoblots corresponding to images in the main text and supplementary figures are shown in Figures S5–S7.

The Glycomics data have been deposited in GlycoPOST (https://glycopost.glycosmos.org/) (77) with the dataset identifier GPST000420 and GPST000418.

## Supporting information

This article contains supporting information.

## Conflict of interests

The authors declare that they have no known competing financial interests or personal relationships that could have appeared to influence the work reported in this paper.
