# Supplementary material for: ZNT5-6 and ZNT7 play an integral role in protein N-glycosylation by supplying Zn2+ to Golgi α-mannosidase II
Source: J Biol Chem. 2024 May 16;300(6):107378. doi: 10.1016/j.jbc.2024.107378 (PMC11209640; doi:10.1016/j.jbc.2024.107378)
Supplement: Supporting Tables and Figures [file mmc1.pdf]

ZNT5-6 and ZNT7 play an integral role in protein *N*-glycosylation by supplying Zn^{2+} to Golgi α-mannosidase II

Hana Yuasa[1,*], Naho Morino[1,*], Takumi Wagatsuma[1,*], Masayuki Munekane[2], Sachiko Ueda[1], Mayu Matsunaga[1], Yasuo Uchida[3], Takane Katayama[1], Toshihiko Katoh[1], Taiho Kambe[1]

*These authors contributed equally to this work.

[1]Division of Integrated Life Science, Graduate School of Biostudies, Kyoto University, Kyoto 606-8502, Japan
[2]Institute of Medical, Pharmaceutical and Health Sciences Faculty of Pharmacy, Kanazawa University, Kanazawa 920-1192, Japan
[3]Department of Molecular Systems Pharmaceutics, Graduate School of Biomedical and Health Sciences, Hiroshima University, 734-0037 Hiroshima city, Japan

TABLE OF CONTENTS

Figure S1. Full-mass profile (*m*/*z* 2500-3500) of permethylated *N*-glycans released by PNGase F from HAP1 WT cells

Figure S2. MS/MS spectra of glycan ions that were specifically increased in HAP-*Z5Z7*-DKO cells

Figure S3. Figure S2. MALDI-TOF/MS analysis of permethylated FNGs

Figure S4. Individual data used in Figure 5B

Figure S5. Full-length images of immunoblots shown in Figure 1

Figure S6. Full-length images of immunoblots shown in Figure 3

Figure S7. Full-length images of immunoblots shown in Figure 4

Table S1. Glycan ion peaks of permethylated *N*-glycans obtained from WT HAP1, HAP-*Z5*-KO (Δ*ZNT5*), HAP-*Z7*-KO (Δ*ZNT7*), and HAP-*Z5Z7*-DKO (Δ*ZNT5*Δ*ZNT7*) cells identified using the MALDI-TOF MS analysis

Table S2. Reliable data of relative protein abundance between MIA-*Z5Z7*-DKO and WT MIA PaCa-2 cells (refer to the Excel file)

Table S3. Increased expression of MGATI, MGATII, and B4GALT1 in HAP-*Z5Z7*-DKO cells in the gene ontology term (biological process) enrichment analysis using the top 50 proteins in the SWATH-MS analysis

Table S4. Increased expression of MGATI, MGATII, and B4GALTs in MIA-*Z5Z7*-DKO cells in the gene ontology term (biological process) enrichment analysis using the top 50 proteins in the SWATH-MS analysis

Table S5. Oligonucleotides used for the generation of sgRNA expression plasmids

Table S6. Primers used for genomic PCR to confirm gene editing in KO cells

Table S7. Ion peaks of permethylated free *N*-glycans obtained from WT HAP1, WT HAP1, HAP-*Z5Z7*-DKO (Δ*ZNT5*Δ*ZNT7*), HAP-*Z4*-KO (Δ*ZNT4*), and HAP-*Z4Z5Z7*-TKO (Δ*ZNT4*Δ*ZNT5*Δ*ZNT7*) cells identified using the MALDI-TOF MS analysis (refer to the Excel file)

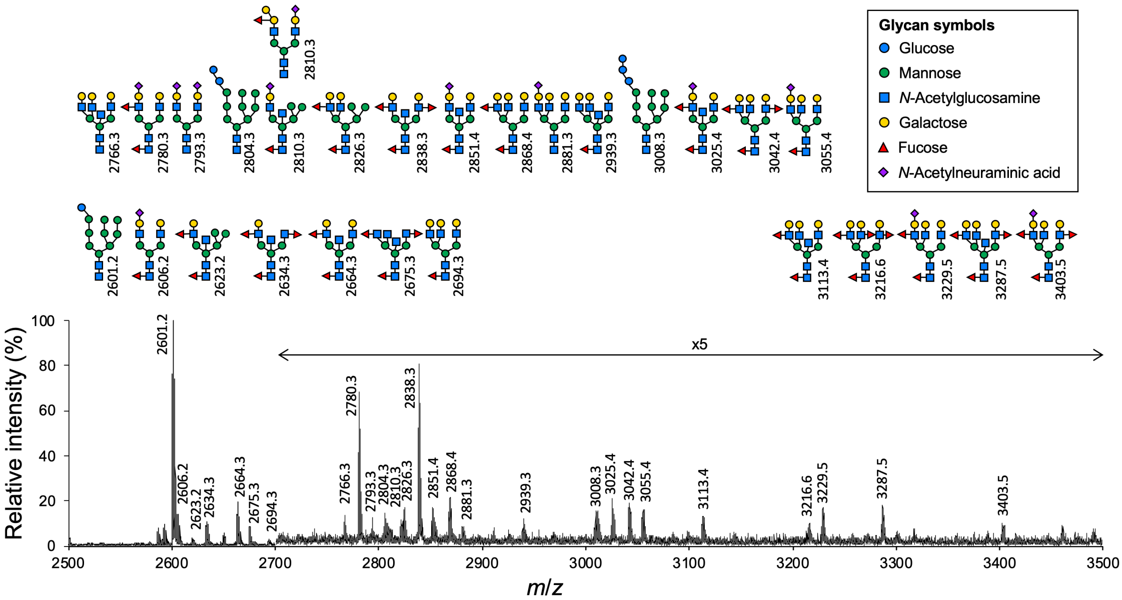

Figure S1. Full-mass profile (*m*/*z* 2500-3500) of permethylated *N*-glycans released by PNGase F from HAP1 WT cells. Representative structures for each glycan ion are shown.

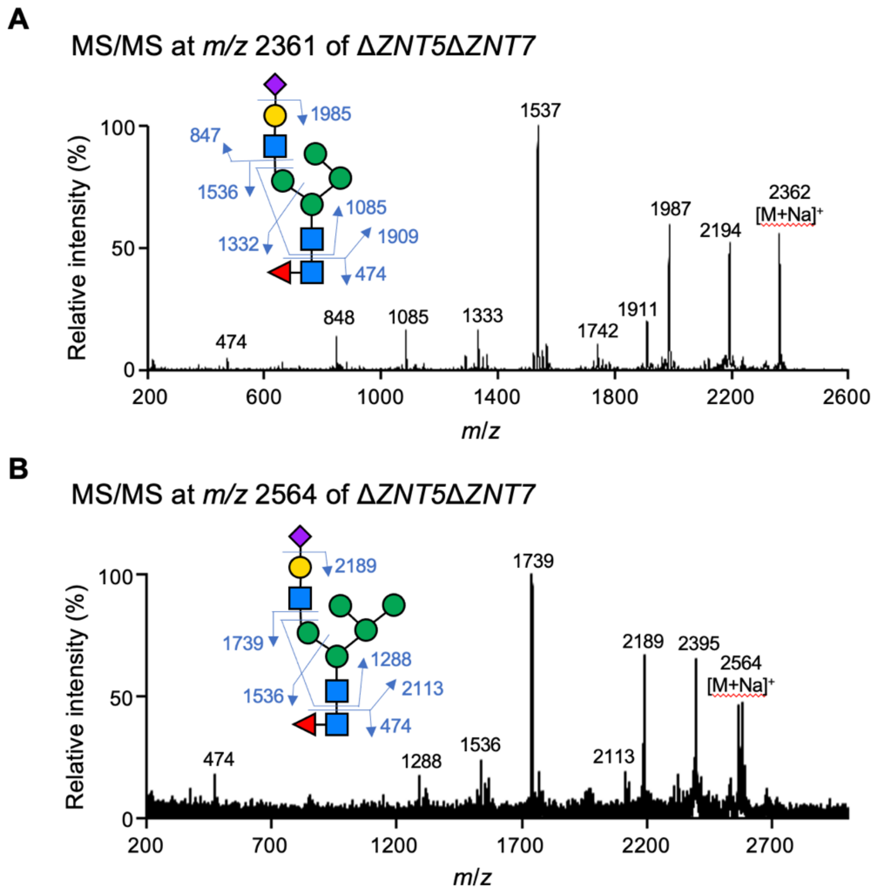

Figure S2. MS/MS spectra of the glycan ions that were specifically increased in HAP-*Z5Z7*-DKO cells. The fragment ions correspond to hybrid-type glycans as illustrated in the figures: $(NeuAc)_1(Gal)_1(GlcNAc)_1(Man)_4(GlcNAc)_2(Fuc)_1$ for *m/z* 2361 (A); $(NeuAc)_1(Gal)_1(GlcNAc)_1(Man)_5(GlcNAc)_2(Fuc)_1$ for *m/z* 2564 (B). Δ*ZNT5*Δ*ZNT7*, HAP-*Z5Z7*-DKO cells.

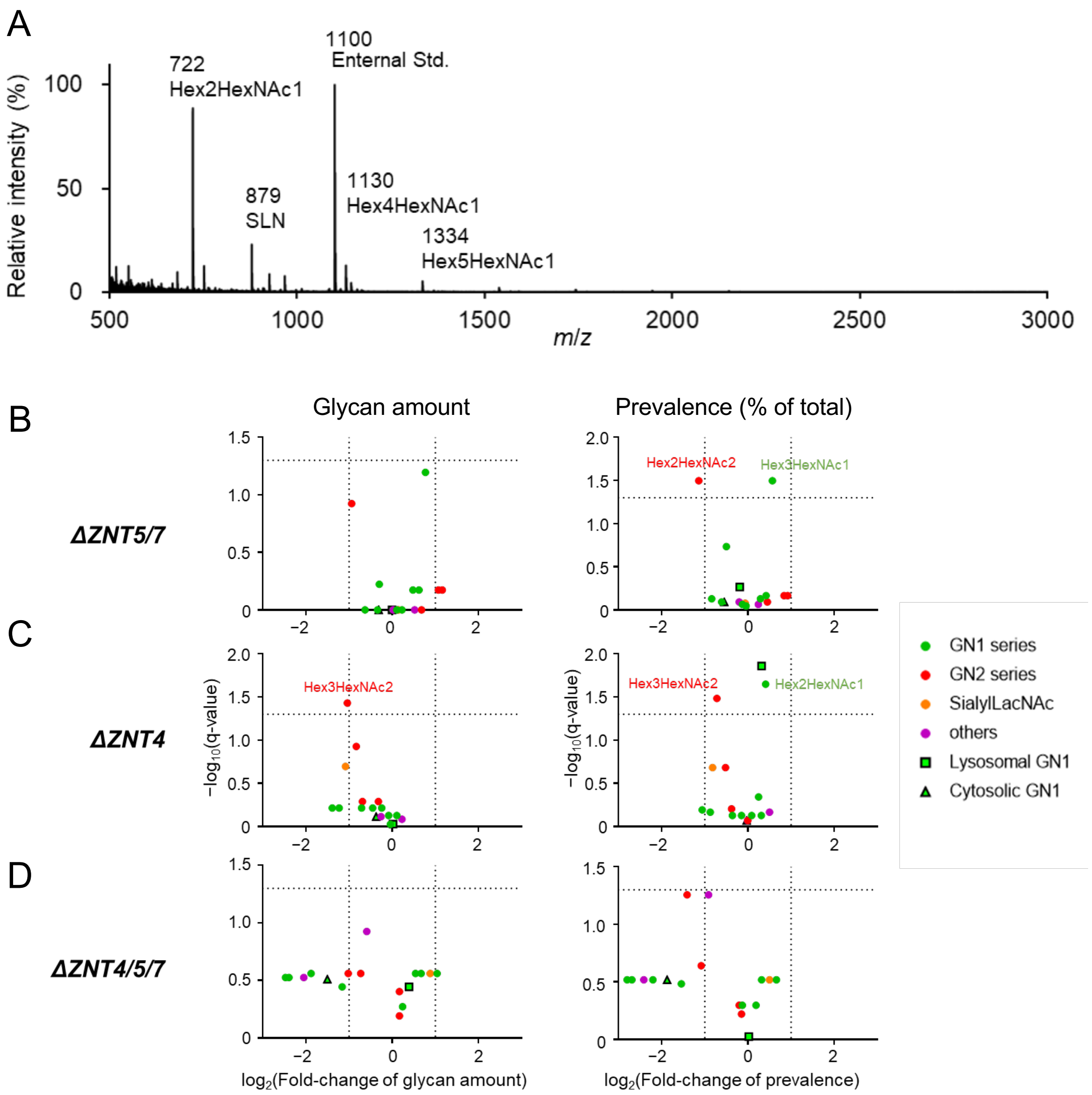

Figure S3. MALDI-TOF/MS analysis of permethylated FNGs. (A) A representative full-MS spectrum of permethylated FNGs from HAP1 WT cells. (B–D) Volcano plots for glycan amount and prevalence (% of the total) of each glycan ion peak for the mutant cells compared with those from HAP1 WT cells. (B) HAP-*Z5Z7*-DKO (Δ*ZNT5/7*) cells. (C) HAP-*Z4*-KO (Δ*ZNT4*) cells. (D) HAP-*Z4Z5Z7*-TKO (Δ*ZNT4/5/7*) cells. In B–D, glycan amount (*left*) and prevalence (% of the total) (*right*) are shown. Glycan types are marked in colors. See Table S7 for more details.

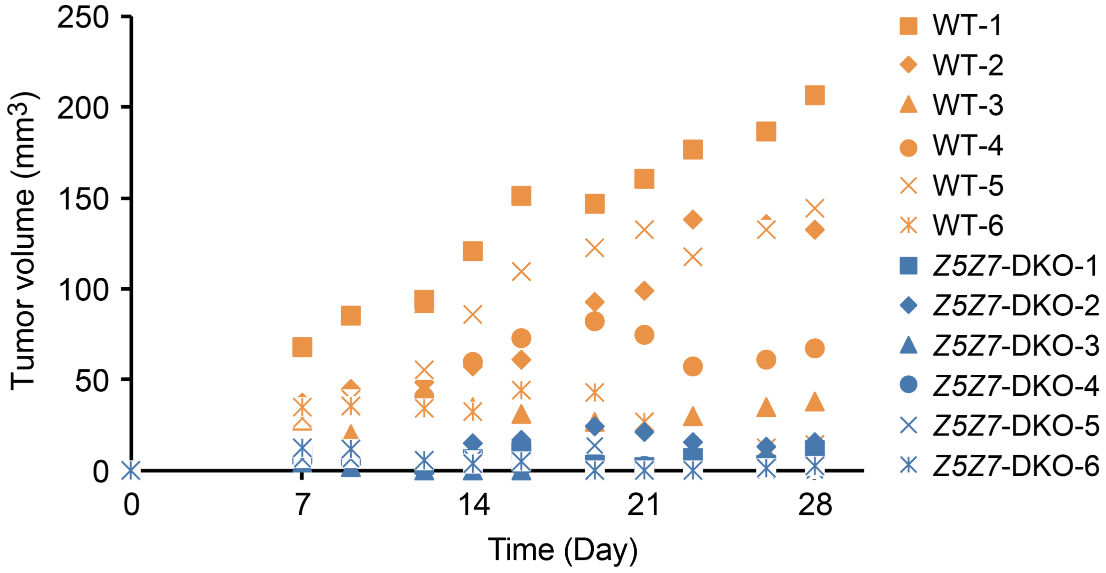

Figure S4. Individual data used in Figure 5B. The individual volume of tumors derived from *s.c.*-inoculated MIA-*Z5Z7*-DKO cells and WT MIA PaCa-2 cells. The indicated symbols indicate the tumor volume of the individual inoculated cells.

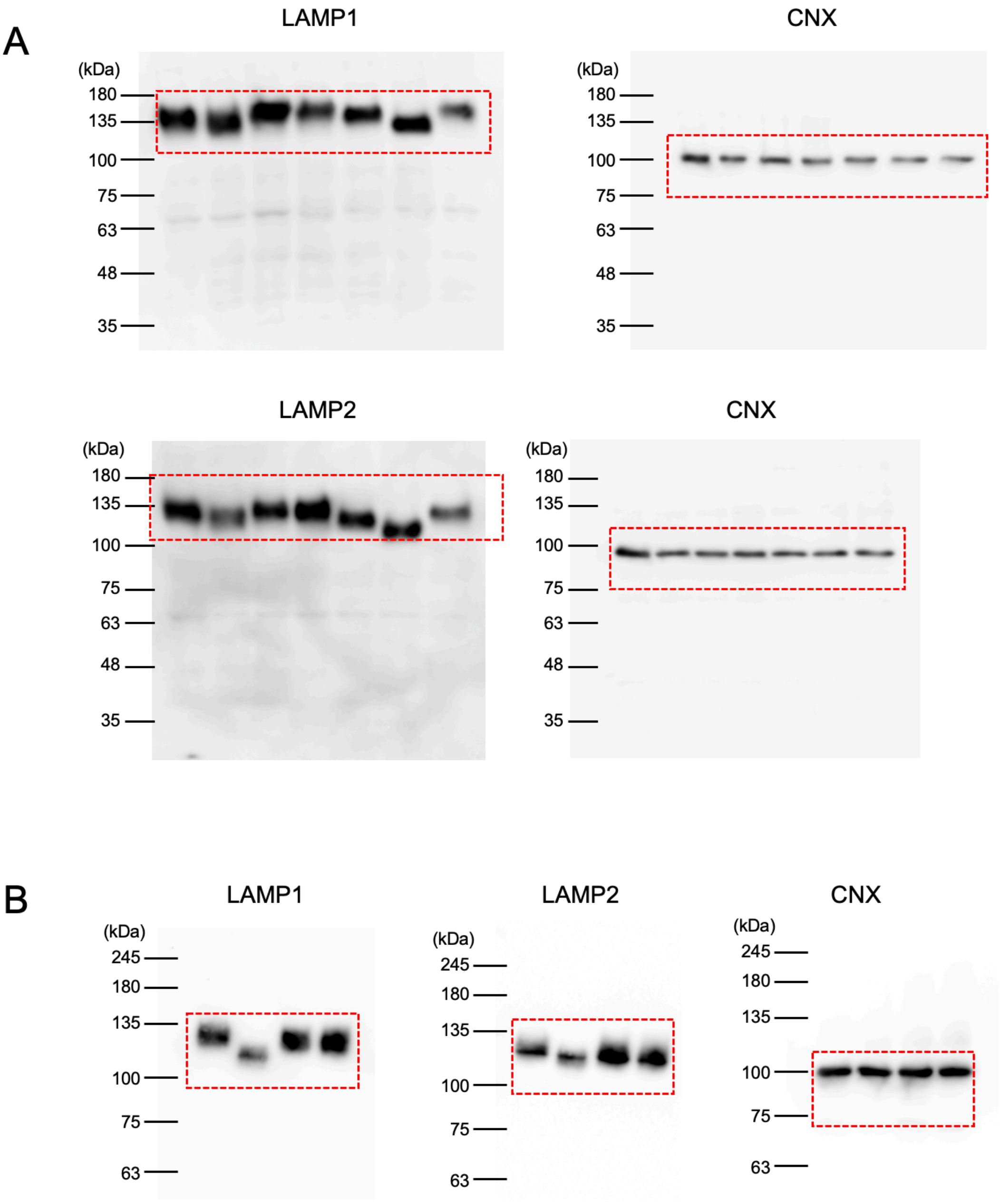
A
LAMP1
(kDa)
180
135
100
75
63
48
35
CNX
(kDa)
180
135
100
75
63
48
35
LAMP2
(kDa)
180
135
100
75
63
48
35
CNX
(kDa)
180
135
100
75
63
48
35
B
LAMP1
(kDa)
245
180
135
100
75
63
LAMP2
(kDa)
245
180
135
100
75
63
CNX
(kDa)
245
180
135
100
75
63

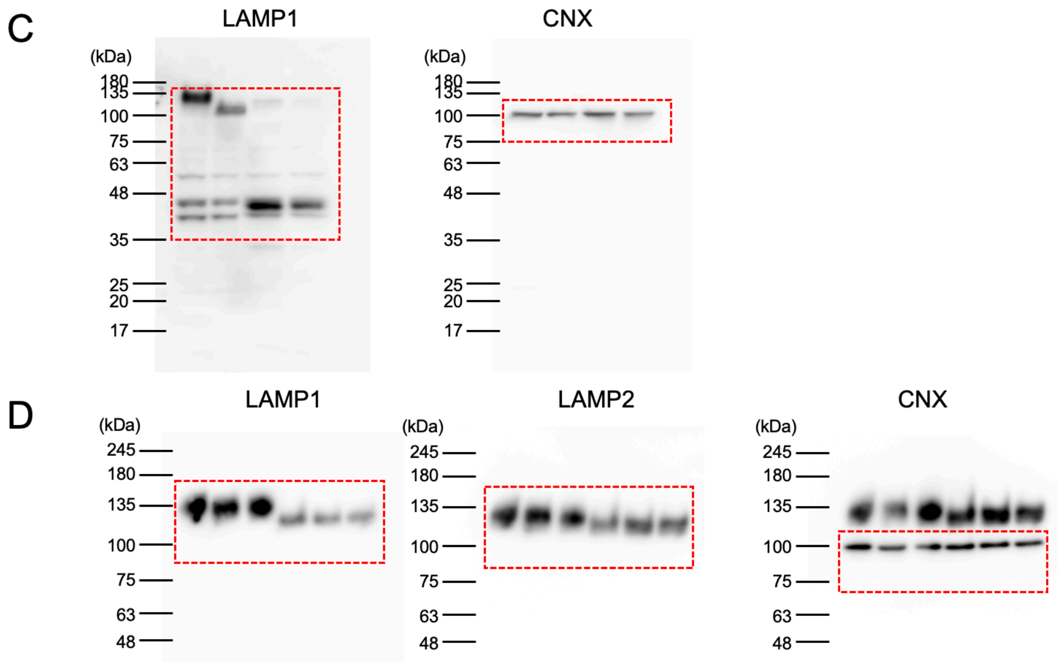

Figure S5. Full-length images of immunoblots shown in Figure 1. The panels used in Figure 1 are boxed. The same blot was used sequentially (after stripping) for detection in each composite figure. The molecular weights of the marker proteins are indicated on the left of the immunoblot images.

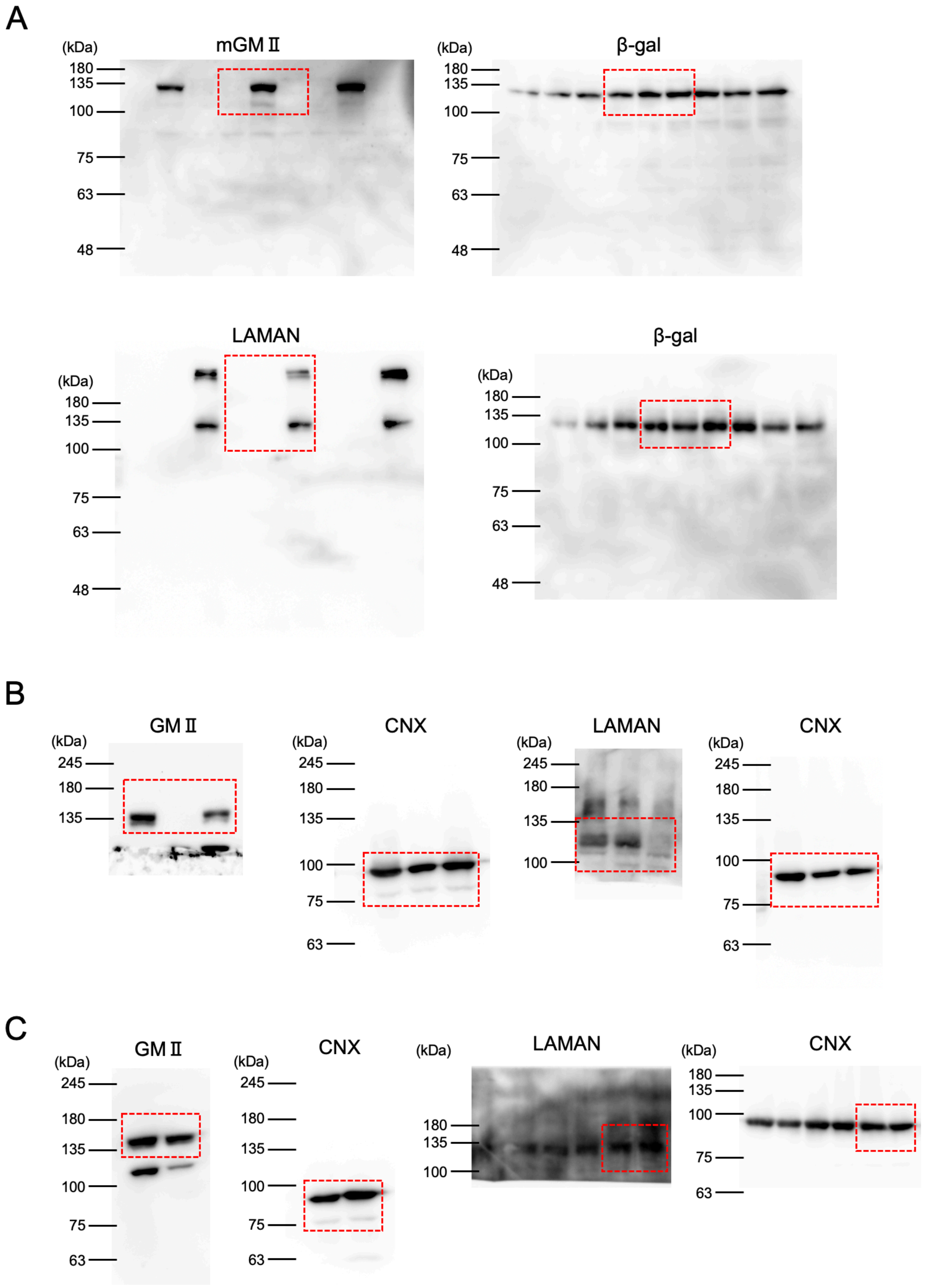
A
mGM II
β-gal
LAMAN
β-gal
B
GM II
CNX
LAMAN
CNX
C
GM II
CNX
LAMAN
CNX
(kDa)
245
180
135
100
75
63
48

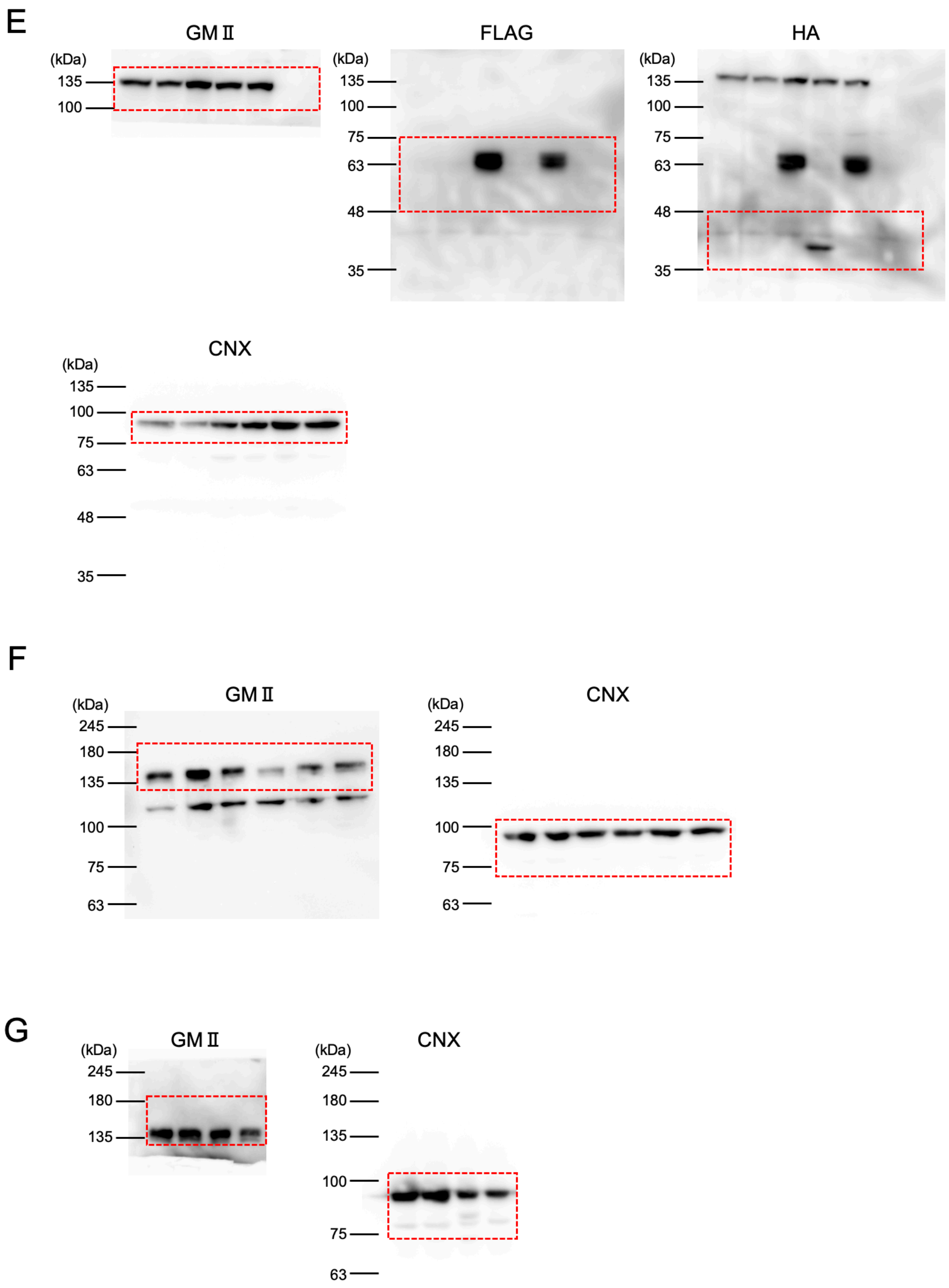

Figure S6. Full-length images of immunoblots used in Figure 3. The panels used in Figure 3 are boxed. The same blot was used sequentially (after stripping) for detection in each composite figure. The molecular weights of the marker proteins are indicated on the left of the immunoblot images.

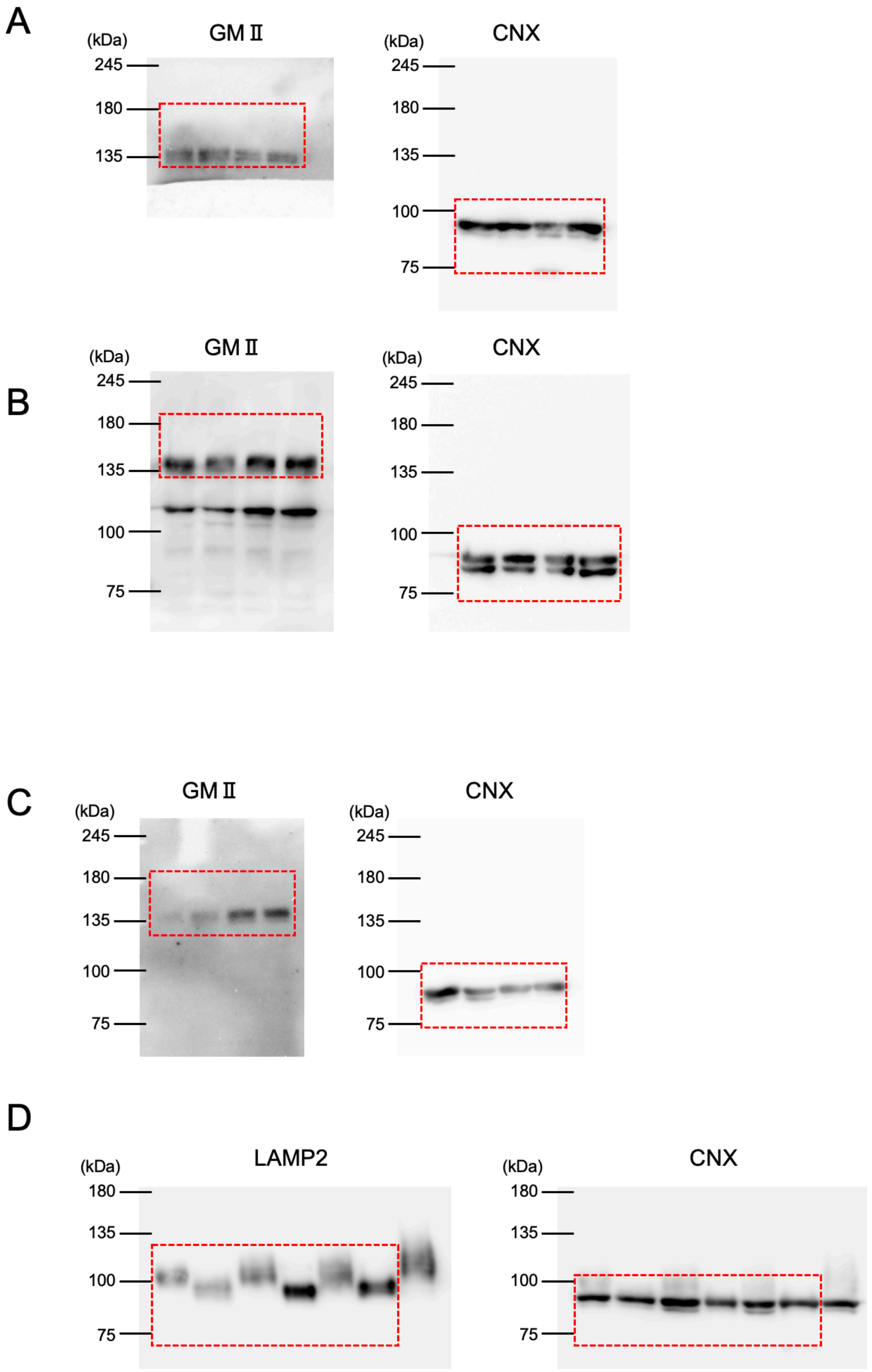

Figure S7. Full-length images of immunoblots used in Figure 4. The panels used in Figure 4 are boxed. The same blot was used sequentially (after stripping) for detection in each composite figure. The molecular weights of the marker proteins are indicated on the left of the immunoblot images.

Table S1. Glycan ion peaks of permethylated *N*-glycans obtained from WT HAP1, HAP-*Z5*-KO (Δ*ZNT5*), HAP-*Z7*-KO (Δ*ZNT7*), and HAP-*Z5Z7*-DKO (Δ*ZNT5*Δ*ZNT7*) cells identified using the MALDI-TOF MS analysis

m/z (Observed)	*m/z* [M + Na]+ (monoisotopic mass)	Deduced glycosyl composition	Glycan type[a]	Prevalence (% to total)[b]						
				WT	Δ*ZNT5*		Δ*ZNT7*		Δ*ZNT5*Δ*ZNT7*	
				Mean ± SD	Mean ± SD	q-value[c]	Mean ± SD	q-value	Mean ± SD	q-value
1171.62	1171.5831	(Hex)3 (HexNAc)2	Pauci-mannose	0.12 ± 0.01	0.27 ± 0.12	0.1663	0.11 ± 0.04	0.8990	0.07 ± 0.01	0.0436
1345.67	1345.6723	(Hex)3 (HexNAc)2 (Deoxyhexose)1	Pauci-mannose	0.14 ± 0.02	0.42 ± 0.24	0.1838	0.13 ± 0.05	0.7240	0.17 ± 0.03	0.4221
1375.75	1375.6828	(Hex)4 (HexNAc)2	Pauci-mannose	0.11 ± 0.03	0.19 ± 0.07	0.2119	0.10 ± 0.06	0.8252	0.27 ± 0.06	0.0569
1416.72	1416.7094	(HexNAc)1 + (Man)3(GlcNAc)2	Complex	0.07 ± 0.02	0.10 ± 0.02	0.3384	0.09 ± 0.04	0.5908	0.03 ± 0.00	0.1078
1579.79	1579.7826	(Hex)2 + (Man)3(GlcNAc)2	High-mannose	6.05 ± 0.11	4.88 ± 1.78	0.4218	3.62 ± 0.92	0.0370	12.54 ± 3.69	0.0937
1590.80	1590.7986	(HexNAc)1 (Deoxyhexose)1 + (Man)3(GlcNAc)2	Complex	0.18 ± 0.06	0.28 ± 0.04	0.1263	0.15 ± 0.05	0.6373	0.17 ± 0.08	0.8990
1620.87	1620.8091	(Hex)1 (HexNAc)1 + (Man)3(GlcNAc)2	Complex/Hybrid	0.09 ± 0.03	0.11 ± 0.05	0.6328	0.10 ± 0.03	0.9406	0.06 ± 0.02	0.2533
1662.80	1661.8357	(HexNAc)2 + (Man)3(GlcNAc)2	Complex	0.08 ± 0.01	0.09 ± 0.02	0.5675	0.07 ± 0.01	0.8252	0.03 ± 0.01	0.0275
1783.87	1783.8824	(Hex)3 + (Man)3(GlcNAc)2	High-mannose	19.79 ± 0.51	16.13 ± 3.38	0.2119	13.31 ± 1.91	0.0209	13.15 ± 0.93	0.0049
1794.91	1794.8984	(Hex)1 (HexNAc)1 (Deoxyhexose)1 + (Man)3(GlcNAc)2	Complex/Hybrid	0.16 ± 0.03	0.12 ± 0.04	0.3913	0.11 ± 0.03	0.2250	0.72 ± 0.15	0.0172
1824.91	1824.9089	(Hex)2 (HexNAc)1 + (Man)3(GlcNAc)2	Hybrid	0.15 ± 0.05	0.15 ± 0.03	0.9497	0.18 ± 0.05	0.5908	0.53 ± 0.08	0.0125
1835.93	1835.9249	(HexNAc)2 (Deoxyhexose)1 + (Man)3(GlcNAc)2	Complex	0.71 ± 0.13	0.78 ± 0.12	0.5903	0.63 ± 0.07	0.5348	0.70 ± 0.34	0.9914
1865.90	1865.9355	(Hex)1 (HexNAc)2 + (Man)3(GlcNAc)2	Complex	0.08 ± 0.02	0.10 ± 0.01	0.3082	0.08 ± 0.04	0.9237	0.04 ± 0.01	0.0868
1987.95	1987.9821	(Hex)4 + (Man)3(GlcNAc)2	High-mannose	8.86 ± 0.38	8.94 ± 1.19	0.9397	7.95 ± 0.45	0.1186	9.51 ± 1.14	0.4919
1999.99	1998.9981	(Hex)2 (HexNAc)1 (Deoxyhexose)1 + (Man)3(GlcNAc)2	Complex/Hybrid	0.19 ± 0.05	0.09 ± 0.00	0.0878	0.14 ± 0.04	0.4105	2.03 ± 0.24	0.0032
2030.01	2029.0087	(Hex)3 (HexNAc)1 + (Man)3(GlcNAc)2	Hybrid	0.17 ± 0.07	0.17 ± 0.04	0.9397	0.25 ± 0.06	0.3341	0.51 ± 0.10	0.0329
2041.00	2040.0247	(Hex)1 (HexNAc)2 (Deoxyhexose)1 + (Man)3(GlcNAc)2	Complex/Hybrid	0.32 ± 0.10	0.20 ± 0.02	0.1979	0.22 ± 0.07	0.3337	0.22 ± 0.03	0.2745
2069.99	2070.0352	(Hex)2 (HexNAc)2 + (Man)3(GlcNAc)2	Complex/Hybrid	0.30 ± 0.10	0.32 ± 0.04	0.7970	0.33 ± 0.06	0.7285	0.31 ± 0.10	0.9397
2081.00	2081.0512	(HexNAc)3 (Deoxyhexose)1 + (Man)3(GlcNAc)2	Complex	0.23 ± 0.04	0.24 ± 0.08	0.9397	0.18 ± 0.01	0.1722	0.35 ± 0.15	0.3571
2173.03	2173.0873	(Hex)2 (HexNAc)1 (Deoxyhexose)2 + (Man)3(GlcNAc)2	Hybrid	0.10 ± 0.02	0.05 ± 0.01	0.0620	0.07 ± 0.01	0.1656	0.66 ± 0.06	0.0029
2187.05	2186.0826	(Hex)2 (HexNAc)1 (NeuAc)1 + (Man)3(GlcNAc)2	Hybrid	0.08 ± 0.02	0.13 ± 0.02	0.1107	0.11 ± 0.01	0.1825	0.45 ± 0.11	0.0207
2192.05	2192.0819	(Hex)5 + (Man)3(GlcNAc)2	High-mannose	19.76 ± 1.01	21.65 ± 1.01	0.1583	22.04 ± 0.10	0.0550	16.53 ± 1.30	0.0738
2204.04	2203.0979	(Hex)3 (HexNAc)1 (Deoxyhexose)1 + (Man)3(GlcNAc)2	Hybrid	0.31 ± 0.03	0.07 ± 0.01	0.0030	0.08 ± 0.01	0.0034	1.79 ± 0.10	0.0026
2244.08	2244.1245	(Hex)2 (HexNAc)2 (Deoxyhexose)1 + (Man)3(GlcNAc)2	Complex/Hybrid	0.79 ± 0.24	0.74 ± 0.15	0.8684	0.94 ± 0.17	0.5066	1.31 ± 0.33	0.1624
2286.11	2285.1510	(Hex)1 (HexNAc)3 (Deoxyhexose)1 + (Man)3(GlcNAc)2	Complex	0.51 ± 0.08	0.40 ± 0.06	0.2119	0.45 ± 0.04	0.3960	0.11 ± 0.02	0.0084
2361.13	2360.1718	(Hex)2 (HexNAc)1 (Deoxyhexose)1 (NeuAc)1 + (Man)3(GlcNAc)2	Hybrid	0.09 ± 0.02	0.15 ± 0.02	0.1006	0.21 ± 0.03	0.0191	4.07 ± 0.85	0.0092
2391.15	2390.1824	(Hex)3 (HexNAc)1 (NeuAc)1 + (Man)3(GlcNAc)2	Hybrid	0.17 ± 0.07	0.26 ± 0.02	0.1798	0.33 ± 0.03	0.0730	1.62 ± 0.37	0.0156
2397.16	2396.1817	(Hex)6 + (Man)3(GlcNAc)2	High-mannose	26.36 ± 0.64	30.79 ± 3.52	0.1731	34.14 ± 1.75	0.0125	16.81 ± 2.21	0.0125
2402.14	2401.1983	(Hex)1 (HexNAc)2 (Deoxyhexose)1 (NeuAc)1 + (Man)3(GlcNAc)2	Complex	0.29 ± 0.03	0.32 ± 0.07	0.5400	0.31 ± 0.03	0.5809	0.37 ± 0.06	0.1805
2419.19	2418.2137	(Hex)2 (HexNAc)2 (Deoxyhexose)2 + (Man)3(GlcNAc)2	Complex/Hybrid	0.92 ± 0.20	0.13 ± 0.01	0.0149	0.31 ± 0.05	0.0275	0.56 ± 0.10	0.1078
2432.17	2431.2089	(Hex)2 (HexNAc)2 (NeuAc)1 + (Man)3(GlcNAc)2	Complex/Hybrid	0.15 ± 0.08	0.31 ± 0.07	0.1202	0.36 ± 0.03	0.0448	0.12 ± 0.05	0.6717
2449.19	2448.2242	(Hex)3 (HexNAc)2 (Deoxyhexose)1 + (Man)3(GlcNAc)2	Hybrid	0.08 ± 0.02	0.05 ± 0.02	0.2110	0.08 ± 0.03	0.9533	1.42 ± 0.37	0.0172
2460.18	2459.2402	(Hex)1 (HexNAc)3 (Deoxyhexose)2 + (Man)3(GlcNAc)2	Complex	0.80 ± 0.10	0.16 ± 0.04	0.0053	0.28 ± 0.06	0.0095	0.07 ± 0.01	0.0032
2490.18	2489.2508	(Hex)2 (HexNAc)3 (Deoxyhexose)1 + (Man)3(GlcNAc)2	Complex	0.63 ± 0.12	0.82 ± 0.21	0.3248	0.94 ± 0.15	0.1078	0.15 ± 0.04	0.0162
2565.20	2564.2716	(Hex)3 (HexNAc)1 (Deoxyhexose)1 (NeuAc)1 + (Man)3(GlcNAc)2	Hybrid	0.05 ± 0.01	0.08 ± 0.02	0.1505	0.12 ± 0.02	0.0210	3.65 ± 0.66	0.0068
2601.19	2600.2815	(Hex)7 + (Man)3(GlcNAc)2	High-mannose	4.26 ± 0.45	4.72 ± 0.99	0.5899	5.15 ± 1.03	0.3373	3.15 ± 0.46	0.0979
2606.21	2605.2981	(Hex)2 (HexNAc)2 (Deoxyhexose)1 (NeuAc)1 + (Man)3(GlcNAc)2	Complex	0.67 ± 0.23	1.51 ± 0.52	0.1219	1.81 ± 0.31	0.0263	0.59 ± 0.18	0.7480
2623.25	2622.3134	(Hex)3 (HexNAc)2 (Deoxyhexose)2 + (Man)3(GlcNAc)2	Hybrid	0.06 ± 0.02	0.04 ± 0.01	0.2533	0.06 ± 0.01	0.8990	1.16 ± 0.23	0.0084
2634.27	2633.3294	(Hex)1 (HexNAc)3 (Deoxyhexose)3 + (Man)3(GlcNAc)2	Complex	0.54 ± 0.06	0.10 ± 0.01	0.0032	0.14 ± 0.04	0.0060	0.03 ± 0.01	0.0029
2653.24	2652.3240	(Hex)4 (HexNAc)2 (Deoxyhexose)1 + (Man)3(GlcNAc)2	Hybrid	0.05 ± 0.01	0.05 ± 0.02	0.7285	0.05 ± 0.02	0.9397	0.24 ± 0.04	0.0084
2664.27	2663.3400	(Hex)2 (HexNAc)3 (Deoxyhexose)2 + (Man)3(GlcNAc)2	Complex	0.94 ± 0.12	0.16 ± 0.06	0.0053	0.16 ± 0.04	0.0048	0.14 ± 0.04	0.0048
2675.29	2674.3560	(HexNAc)4 (Deoxyhexose)3 + (Man)3(GlcNAc)2	Complex	0.43 ± 0.04	0.19 ± 0.05	0.0174	0.18 ± 0.06	0.0172	0.02 ± 0.01	0.0029
2694.27	2693.3506	(Hex)3 (HexNAc)3 (Deoxyhexose)1 + (Man)3(GlcNAc)2	Complex	0.18 ± 0.07	0.25 ± 0.08	0.4415	0.37 ± 0.08	0.0809	0.06 ± 0.02	0.0992
2766.29	2764.3877	(Hex)3 (HexNAc)4 + (Man)3(GlcNAc)2	Complex	0.13 ± 0.03	0.06 ± 0.02	0.0979	0.11 ± 0.03	0.4218	0.06 ± 0.02	0.0730
2780.31	2779.3873	(Hex)2 (HexNAc)2 (Deoxyhexose)2 (NeuAc)1 + (Man)3(GlcNAc)2	Complex	0.66 ± 0.21	0.11 ± 0.04	0.0358	0.17 ± 0.02	0.0466	0.30 ± 0.07	0.1006
2793.31	2792.3826	(Hex)2 (HexNAc)2 (NeuAc)2 + (Man)3(GlcNAc)2	Complex	0.21 ± 0.13	0.08 ± 0.04	0.2425	0.10 ± 0.02	0.3267	0.05 ± 0.02	0.1805
2797.31	2796.4026	(Hex)3 (HexNAc)2 (Deoxyhexose)3 + (Man)3(GlcNAc)2	Complex/Hybrid	0.03 ± 0.01	0.04 ± 0.02	0.8095	0.04 ± 0.01	0.4919	0.76 ± 0.14	0.0081
2804.26	2804.3814	(Hex)8 + (Man)3(GlcNAc)2	High-mannose	0.10 ± 0.02	0.06 ± 0.01	0.1505	0.08 ± 0.02	0.4218	0.06 ± 0.02	0.1965
2810.30	2809.3979	(Hex)3 (HexNAc)2 (Deoxyhexose)1 (NeuAc)1 + (Man)3(GlcNAc)2	Complex/Hybrid	0.07 ± 0.01	0.05 ± 0.02	0.4218	0.06 ± 0.01	0.5809	0.94 ± 0.31	0.0310
2826.33	2826.4132	(Hex)4 (HexNAc)2 (Deoxyhexose)2 + (Man)3(GlcNAc)2	Hybrid	0.05 ± 0.01	0.03 ± 0.00	0.0532	0.03 ± 0.00	0.0591	0.19 ± 0.04	0.0172
2838.33	2837.4292	(Hex)2 (HexNAc)3 (Deoxyhexose)3 + (Man)3(GlcNAc)2	Complex	0.78 ± 0.05	0.16 ± 0.05	0.0029	0.12 ± 0.02	0.0026	0.15 ± 0.05	0.0029
2851.37	2850.4244	(Hex)2 (HexNAc)3 (Deoxyhexose)1 (NeuAc)1 + (Man)3(GlcNAc)2	Complex	0.17 ± 0.06	0.88 ± 0.48	0.1263	0.49 ± 0.14	0.0600	0.08 ± 0.04	0.1587
2868.35	2867.4398	(Hex)3 (HexNAc)3 (Deoxyhexose)2 + (Man)3(GlcNAc)2	Complex	0.22 ± 0.05	0.11 ± 0.04	0.1006	0.12 ± 0.02	0.0854	0.08 ± 0.04	0.0588
2881.32	2880.4350	(Hex)3 (HexNAc)3 (NeuAc)1 + (Man)3(GlcNAc)2	Complex	0.07 ± 0.03	0.17 ± 0.08	0.1805	0.18 ± 0.04	0.0532	0.03 ± 0.01	0.1187
2939.35	2938.4769	(Hex)3 (HexNAc)4 (Deoxyhexose)1 + (Man)3(GlcNAc)2	Complex	0.12 ± 0.03	0.18 ± 0.09	0.4542	0.28 ± 0.13	0.1798	0.02 ± 0.01	0.0259
2968.34	2966.4718	(Hex)2 (HexNAc)2 (Deoxyhexose)1 (NeuAc)2 + (Man)3(GlcNAc)2	Complex	0.05 ± 0.01	0.13 ± 0.07	0.1805	0.15 ± 0.03	0.0164	0.04 ± 0.02	0.4919
2984.38	2983.4871	(Hex)3 (HexNAc)2 (Deoxyhexose)2 (NeuAc)1 + (Man)3(GlcNAc)2	Hybrid	0.01 ± 0.01	0.02 ± 0.01	0.8684	0.02 ± 0.01	0.4528	0.26 ± 0.08	0.0243
3008.34	3008.4810	(Hex)9 + (Man)3(GlcNAc)2	High-mannose	0.11 ± 0.02	0.10 ± 0.04	0.6915	0.09 ± 0.02	0.4635	0.07 ± 0.01	0.0700
3025.41	3024.5137	(Hex)2 (HexNAc)3 (Deoxyhexose)2 (NeuAc)1 + (Man)3(GlcNAc)2	Complex	0.22 ± 0.07	0.05 ± 0.03	0.0591	0.04 ± 0.01	0.0416	0.08 ± 0.03	0.0902
3042.37	3041.5290	(Hex)3 (HexNAc)3 (Deoxyhexose)3 + (Man)3(GlcNAc)2	Complex	0.17 ± 0.02	0.10 ± 0.03	0.0979	0.14 ± 0.04	0.4530	0.07 ± 0.04	0.0516
3055.40	3054.5242	(Hex)3 (HexNAc)3 (Deoxyhexose)1 (NeuAc)1 + (Man)3(GlcNAc)2	Complex	0.20 ± 0.09	0.58 ± 0.30	0.1805	0.81 ± 0.16	0.0200	0.08 ± 0.04	0.1740
3113.44	3112.5661	(Hex)3 (HexNAc)4 (Deoxyhexose)2 + (Man)3(GlcNAc)2	Complex	0.13 ± 0.02	0.08 ± 0.07	0.3510	0.08 ± 0.02	0.1006	0.03 ± 0.01	0.0084
3143.47	3142.5766	(Hex)4 (HexNAc)4 (Deoxyhexose)1 + (Man)3(GlcNAc)2	Complex	0.06 ± 0.01	0.09 ± 0.05	0.4542	0.24 ± 0.14	0.1583	0.02 ± 0.02	0.1203
3216.59	3215.6182	(Hex)3 (HexNAc)3 (Deoxyhexose)4 + (Man)3(GlcNAc)2	Complex	0.08 ± 0.00	0.03 ± 0.01	0.0084	0.04 ± 0.00	0.0029	0.04 ± 0.01	0.0263
3229.49	3228.6134	(Hex)3 (HexNAc)3 (Deoxyhexose)2 (NeuAc)1 + (Man)3(GlcNAc)2	Complex	0.17 ± 0.06	0.09 ± 0.07	0.3029	0.09 ± 0.02	0.1674	0.05 ± 0.03	0.1006
3287.51	3286.6553	(Hex)3 (HexNAc)4 (Deoxyhexose)3 + (Man)3(GlcNAc)2	Complex	0.16 ± 0.02	0.07 ± 0.03	0.0427	0.06 ± 0.02	0.0174	0.03 ± 0.01	0.0032
3403.48	3402.7026	(Hex)3 (HexNAc)3 (Deoxyhexose)3 (NeuAc)1 + (Man)3(GlcNAc)2	Complex	0.08 ± 0.03	0.04 ± 0.01	0.1798	0.04 ± 0.01	0.1969	0.04 ± 0.01	0.1505

a, Glycan types are classified based on the deduced glycan compositions. Complex/Hybrid may possibly contain both structures.

b, Prevalences, percentages to the total identified glycan ion intensity, are shown by means ± standard deviation (SD) of the experiments with the biological triplicates (n = 3).

c, Multiple *t*-test with false discovery rate correction (Benjamini & Hochberg, 1995) with Q = 5 % was used for statistical comparison (vs. WT). P values were adjusted to q-values.

Table S2. Reliable data of relative protein abundance between MIA-*Z5Z7*-DKO and WT MIA PaCa-2 cells (refer to the Excel file)

Table S3. Increased expression of MGATI, MGATII, and B4GALT1 in HAP-*Z5Z7*-DKO cells in the SWATH-MS analysis

Rank	Name	DKO/WT	p-value
10	MGAT2	2.675658842	0.00221
15	B4GALT1	2.31226041	0.007882365
41	MGAT1	1.993736969	0.111468325

Table S4. Increased expression of MGATI, MGATII, and B4GALTs in MIA-*Z5Z7*-DKO cells in the SWATH-MS analysis

Rank	Name	DKO/WT	p-value
3	MGAT2	3.056217461	0.000260531
5	B4GALT7	2.840182378	0.000113723
10	B4GALT1	2.724308269	0.001262725
18	MGAT1	2.450870522	0.000622631

Table S5. Oligonucleotides used for the generation of sgRNA expression plasmids

**Gene**	**Forward primer (5′ to 3′)**	**Reverse primer (5′ to 3′)**
ZIP8	CACCG<u>TCAACATAACTGTCGACTTT</u>	AAACAAAGTCGACAGTTATGTTGAC
ZIP9	CACCG<u>TTGGTGGGATGTTACGTGGC</u>	AAACGCCACGTAACATCCCACCAAC
ZIP14	CACCG<u>TCTAATACATCGGTATGGC</u>	AAACGCCATACCGATGTATTAGAC
TMEM165	CACCG<u>TCCAGGGAACGGCCGCGCAT</u>	AAACATGCGCGGCCGTTCCCTGGAC
SPAC1	CAAC<u>GCATACACTTGCCCGAGACT</u>	AAACAGTCTCGGGCAAGTGTATGC
GMII	CACCG<u>AGATCTCTTACCTTTCAAAG</u>	AAACCTTTGAAAGGTAAGAGATCTC
LAMAN	CACCG<u>CCTCACACACATGATGACG</u>	AAACCGTCATCATGTGTGTGAGGC

Guide RNA sequences are underlined.

Table S6. Primers used for genomic PCR to confirm gene editing in KO cells

Gene	Forward primer (5′ to 3′)	Reverse primer (5′ to 3′)
ZIP8	GGATGTGAAGAGAGACTTAGAACATTGGTG	ACTCACAAGCCTAATAACTGGACCATTCTC
ZIP9	GCCACTGGAAATTTGTTGTCTAGTGGTTG	AACTAGGGAAGCAGCTTCCCTTTGCAACT
ZIP14	AGAGACTGAGGGATAGTCTCAACA	CTTACCGTGGAGAGGTTCCTGTGTC
TMEM165	CCTCCCGGATGGTGCTGACTGCTCCCTAAG	TTGCCCGCTCCTTTAGGGAAAGGGAGAGGG
SPAC1	GATGGTCTCGATCTCCTGACCTCGTGATC	ACCATGTCCCAACAGAGTTTCATACACTGG
GMII	GGTCCTAAAGCTGAAAGAAGACTCACGGAG	CCCCCAAGGCAGAGTCTTGCACTGTTACCC
LAMAN	TCCCGGAGTGTGAATGAGAGCTGCCCTGTG	GATGTACTGCACACCGGCGTGCTGGATGTC

Table S7. Ion peaks of permethylated free *N*-glycans obtained from WT HAP1, WT HAP1, HAP-*Z5Z7*-DKO (Δ*ZNT5*Δ*ZNT7*), HAP-*Z4*-KO (Δ*ZNT4*), and HAP-*Z4Z5Z7*-TKO (Δ*ZNT4*Δ*ZNT5*Δ*ZNT7*) cells identified using the MALDI-TOF MS analysis (refer to the Excel file)
